# Supplementary figures and images for: Comparative transcriptome analysis in two contrasting genotypes for Sclerotinia sclerotiorum resistance in sunflower
Source: PLoS One. 2024 Dec 19;19(12):e0315458. doi: 10.1371/journal.pone.0315458 (PMC11658501; doi:10.1371/journal.pone.0315458)

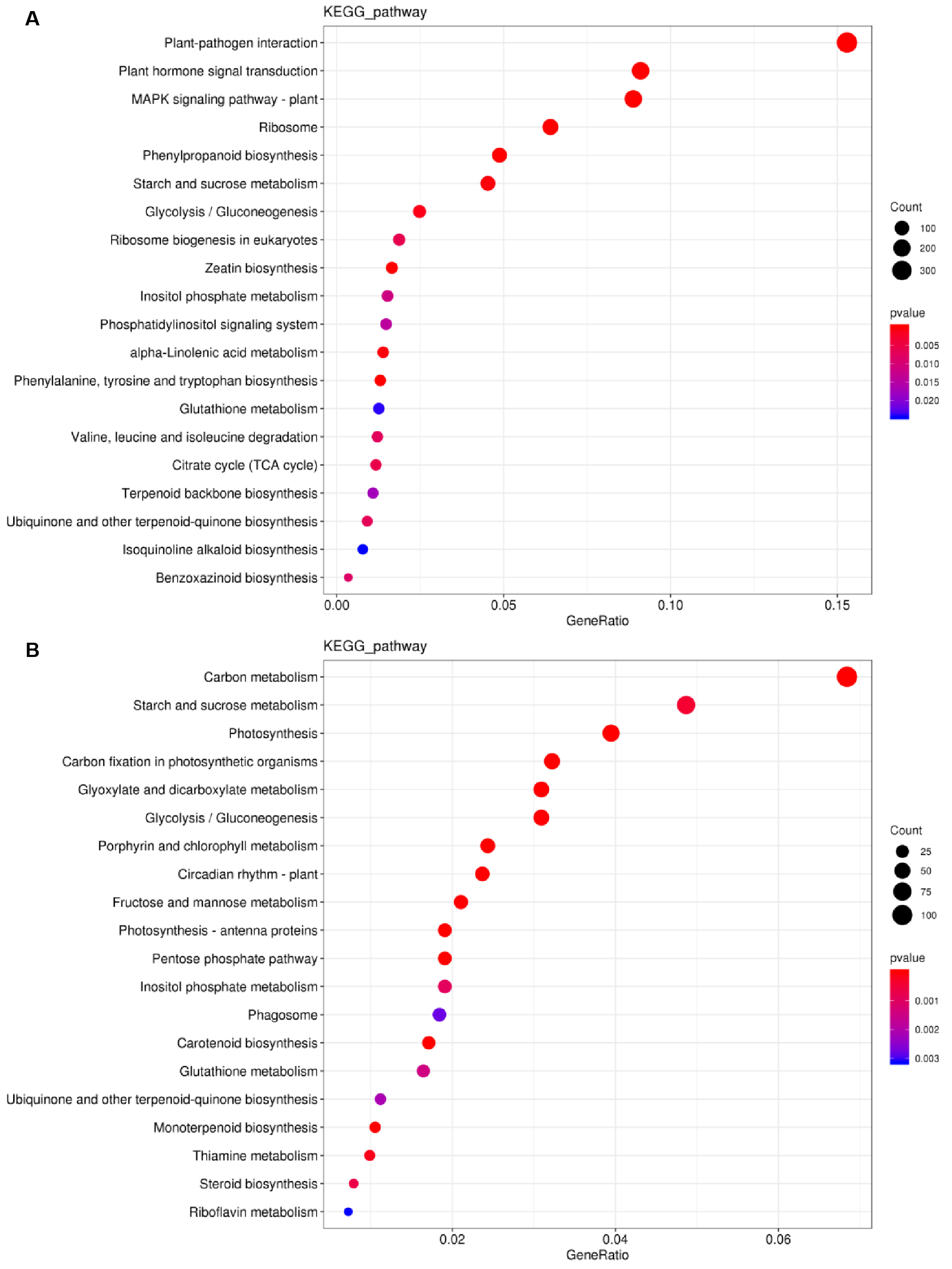

Supplement: S1 Fig — Kyoto Encyclopedia of Genes and Genome (KEGG) analysis for up- (A) and down-regulated (B) DEGs in DS genotype, respectively. (TIF) [file pone.0315458.s016.tif]

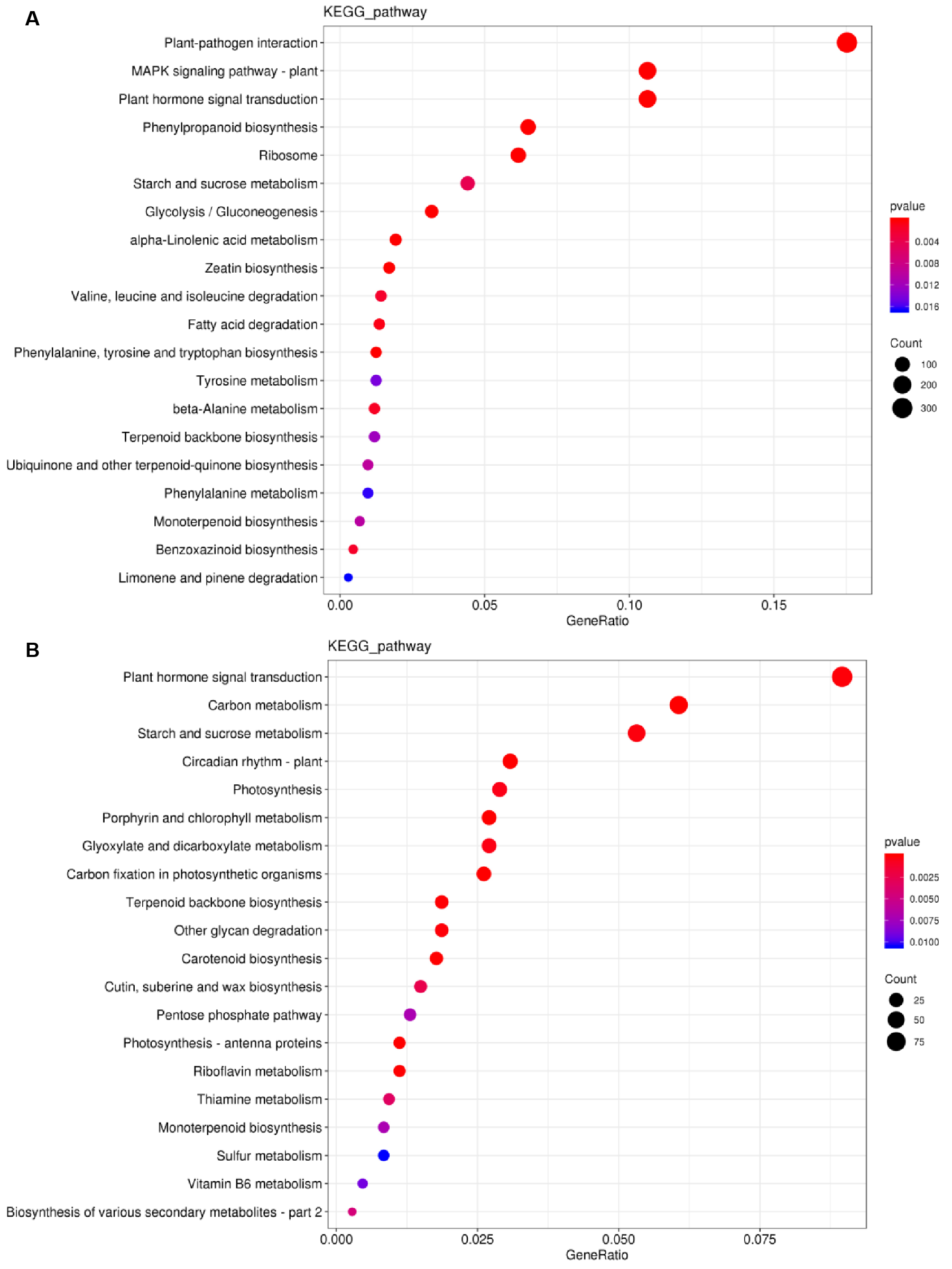

Supplement: S2 Fig — Kyoto Encyclopedia of Genes and Genome (KEGG) analysis for up- (A) and down-regulated (B) DEGs in DR genotype, respectively. (TIF) [file pone.0315458.s017.tif]
