# Supplementary material for: Comparative transcriptome analysis in two contrasting genotypes for Sclerotinia sclerotiorum resistance in sunflower
Source: PLoS One. 2024 Dec 19;19(12):e0315458. doi: 10.1371/journal.pone.0315458 (PMC11658501; doi:10.1371/journal.pone.0315458)
Supplement: Attachment — Submitted filename: PONE-D-23-14214_R2_reviewer_R1_19_6_24.pdf [file pone.0315458.s020.pdf]

PLOS ONE

Comparative Transcriptome Analysis in Two Contrasting Genotypes for Sclerotinia Sclerotiorum Resistance in Sunflower

--Manuscript Draft--

**Manuscript Number:**	PONE-D-23-14214R2
**Article Type:**	Research Article
**Full Title:**	Comparative Transcriptome Analysis in Two Contrasting Genotypes for Sclerotinia Sclerotiorum Resistance in Sunflower
**Short Title:**	Transcriptome response to Sclerotinia sclerotiorum in sunflower
**Corresponding Author:**	Jingang Liu<br>Liaoning Academy of Agricultural Sciences<br>Shenyang, CHINA
**Keywords:**	Differentially expressed genes; Molecular mechanisms; Sunflower (Helianthus annuus); Sclerotinia sclerotiorum; Transcriptomic
**Abstract:**	Sclerotinia sclerotiorum as a necrotrophic fungus causes the devastating diseases in many important oilseed crops worldwide. The preferred strategy for controlling S. sclerotiorum is to develop resistant varieties, but the molecular mechanisms underlying S. sclerotiorum resistance remain poorly defined in sunflower (Helianthus annuus). Here, a comparative transcriptomic analysis was performed in leaves of two contrasting sunflower genotypes, disease susceptible (DS) B728 and disease resistant (DR) C6 after S. sclerotiorum inoculation. At 24 h post-inoculation, the DR genotype exhibited no visible growth of the hyphae as well as greater activity of superoxide dismutase activity (SOD), peroxidase (POD), catalase (CAT), glutathione-S-transferase (GST), ascorbate peroxidase (APX) and monodehydroascorbate reductase (MDAR) than DS genotype. A total of 10151 and 7439 differentially expressed genes (DEGs) were detected in DS and DR genotypes, respectively. Most of DEGs were enriched in cell wall organisation, protein kinase activity, hormone, transcription factor activities, redox homeostasis, immune response, and secondary metabolism. Differential expression of genes involved in expansins, pectate lyase activities, ethylene biosynthesis and signaling and antioxidant activity after S. sclerotiorum infection could potentially be responsible for the differential resistance among two genotypes. In summary, these finding provide additional insights into the potential molecular mechanisms of S. sclerotiorum's defense response and facilitate the breeding of Sclerotinia-resistant sunflower varieties.
**Order of Authors:**	Mingzhu Zhao
	Bing Yi
	Xiaohong Liu
	Dexing Wang
	Dianxiu Song
	Enyu Sun
	Liangji Cui
	Jingang Liu
	Liangshan Feng
**Response to Reviewers:**	Reviewer #2:<br>Please add the NCBI BioProject ID ‘PRJNA908908’ in the manuscript.<br>Response: All the raw data of the transcriptome sequence have been deposited in the NCBI BioProject database (https://www.ncbi.nlm.nih.gov/bioproject/PRJNA908908) under the BioProject PRJNA908908.<br><br>According to figure 4 B-E (figure 6 in previous version), nearly most genes are differentially expressed in both DS and DR genotypes (|log2(FC)| > 1), although the fold change varied. The expression of these genes was not statistically compared

between DS and DR genotypes to narrow down the candidates, which could have been inferred from this data.
Response: The expression of these genes was compared between DS and DR genotypes, which were shown in Fig. 4(A-D), and the results section.

The read cleaning method is unclear and irreproducible. For example, a read should not be ‘annotated based on the reference genome’. This revised method section still contains many errors, making the results unreliable.
Response: Raw reads of fastq format were firstly processed through in-house perl scripts. In this step, the clean reads were obtained by removing adapter-containing reads, ploy-N-containing reads, and low quality reads from raw data. The Q20, Q30, GC-content and sequence duplication level of the clean data were calculated. All the downstream analyses were based on clean data with high quality. The adaptor sequences and low-quality sequence reads were removed from the data sets. Raw sequences were transformed into clean reads after data processing. These clean reads were then mapped to the reference genome sequence. Only reads with a perfect match or one mismatch were further analyzed and annotated based on the sunflower reference genome. The HanXRQr1.0 genome was used as reference in the present study. Then, Hisat2 tools soft were used to map with reference genome. The StringTie Reference Annotation Based Transcript (RABT) assembly method was used to construct and identify both known and novel transcripts from Hisat2 alignment results.

The reference genome information is still not provided, making it impossible to assess the results. Not only the ‘new genes’, but also the ‘reference genes’ have only an ‘ID’ available, which largely limits the contribution to the community.
Response: The HanXRQr1.0 genome was used as reference in the present study.

The numbers of expressed genes were previously 22,077 and 22,579, but in the present version, they increased a lot. Again, without the information of the reference genome, it is impossible to know what happened.
Response: After careful inspection again，in the present version, the numbers of expressed genes were false, and those in the previous version were correct.

Without sufficient data, I cannot comment on the numbers listed.

Reviewer #3:
Authors have conducted transcriptome analysis of a resistant and a susceptible genotype of sunflower against Sclerotinia sclerotiorum. I have some suggestions and comments, which are given below. Page numbers are based on the revised, track-change version.
Major comments:
1) Figure-4, S1, and S2 are not legible. Hence, I couldn’t check the information associated with these figures. These figures need to be updated.
Response: These figures were modified.

2) One major criticism is that all the follow-up analyses were done with the DEGs (Differentially Expressed Genes) upon infection in both the resistant and the susceptible genotypes. This was pointed out by one of the reviewers. However, the author didn’t do a good job of resolving the issue. Ideally, it would be better to focus on DEGs that are unique to either the resistant or the susceptible genotype. Alternatively, performing clustering of DEGs could have been better.
Response: In GO analysis, the unique up- or down-regulated DEGs in either DS genotype B728 or DR genotype C6.

3) English is very poor in some instances. For example, on page 17, lines 2-3, sentence construction is not correct. The same is true for line-8-9. There are multiple examples like this throughout the manuscript. Correcting those will improve the readability of the manuscript.
Response: According the suggestion, these sentences were modified.

4) Did you use any filtering to get rid of the low-expressed genes, otherwise there will be DEGs that were expressed barely.

	Response: Genes with low expression (FPKM <1) will be eliminated by default in the analysis process.<br><br># Minor comments:<br>1) Usage of abbreviations should be kept at a minimum level. CWDE for 'cell wall degrading enzyme' (page 3, line-11) is unacceptable.<br>Response: Many abbreviations were deleted in the revised manuscript, such as for oxalic acid, cell-wall-degrading enzymes, programmed cell death, systemic acquired resistance, programmed cell death, pathogenesis related proteins, pathogen-associated molecular pattern.<br><br>2) Page-5, line-6, why 24 hpi was used for the experiment?<br>Response: As the visible lesions appeared in DS genotype rather than DR genotype at 24 h post-inoculation, then the 20 leaves samples within the range of 0.5 cm bordering the extending lesion from inoculated and mock-inoculated plants were harvested for each replicate.<br><br>3) Page 6, line 20, how the raw reads were processed?<br>Response: Raw reads of fastq format were firstly processed through in-house perl scripts. In this step, the clean reads were obtained by removing adapter-containing reads, ploy-N-containing reads, and low quality reads from raw data. The Q20, Q30, GC-content and sequence duplication level of the clean data were calculated. All the downstream analyses were based on clean data with high quality.<br><br>4) What kind of analysis was performed using MapMan? MapMan is a software, not an analysis.<br>Response: According the suggestion, the MapMan analysis was modified as MapMan software.<br><br>5) Page 8, line 27, what are the criteria for being expressed?<br>Response: FPKM>1.<br><br>6) GO analysis of all DEGs is meaningless, authors should have focused on unique DEGs.<br>Response: The GO analysis of all DEGs was deleted in the revised manuscript.<br><br>7) Page-9, line-23, "In the CC category, "cell", "cell part"……were most frequent". Is this kind of information meaningful?<br>Response: The GO analysis of all DEGs was deleted in the revised manuscript.<br><br>8) Common GOs for both genotypes represented in Figure-3, are not very informative.<br>Response: The Figure-3 in previous version was deleted, and the S7-8 Tables were added in the present version. The S7-8 Tables were the GO analysis to identify enriched gene ontologies among the common DEGs in DS and DR genotypes.<br><br>9) Page 10, lines 11-18, describe GO terms associated with the unique DEGs for each genotype. This section is useful. Authors should expand this section.<br>Response: According the suggestion, the unique up- and down-regulated DEGs in DS genotype B728 were used for GO analysis, which were also shown in S9-10 Tables.<br><br>10) The result section for the analysis using MapMan is very rudimentary.<br>Response: According another reviewer's suggestion, in the pathway analysis of DEGs using MapMan, the expressions of these genes involved in plant hormone signaling pathways, redox homeostasis and cell wall organisation were compared between DS and DR genotypes, which were shown in Fig. 4(A-D), and the results section.
**Additional Information:**	
**Question**	**Response**
**Financial Disclosure**<br><br>Enter a financial disclosure statement that describes the sources of funding for the	The study was supported by China Agriculture Research System of MOF and MARA (CARS-14), and Shenyang Seed Industry Innovation Project (22-318-2-19). The funders had no role in study design, data collection and analysis, decision to publish, or preparation of the manuscript.

Powered by Editorial Manager® and ProduXion Manager® from Aries Systems Corporation

work included in this submission. Review the submission guidelines for detailed requirements. View published research articles from *PLOS ONE* for specific examples.<br><br>This statement is required for submission and **will appear in the published article** if the submission is accepted. Please make sure it is accurate.<br><br>**Unfunded studies**<br>Enter: *The author(s) received no specific funding for this work.*<br><br>**Funded studies**<br>Enter a statement with the following details:<br>• Initials of the authors who received each award<br>• Grant numbers awarded to each author<br>• The full name of each funder<br>• URL of each funder website<br>• Did the sponsors or funders play any role in the study design, data collection and analysis, decision to publish, or preparation of the manuscript?<br>• **NO** - Include this sentence at the end of your statement: *The funders had no role in study design, data collection and analysis, decision to publish, or preparation of the manuscript.*<br>• **YES** - Specify the role(s) played.<br><br>* typeset	
**Competing Interests**<br><br>Use the instructions below to enter a competing interest statement for this submission. On behalf of all authors, disclose any competing interests that could be perceived to bias this work—acknowledging all financial support and any other relevant financial or non-financial competing interests.<br><br>This statement is required for submission and **will appear in the published article** if the submission is accepted. Please make sure it is accurate and that any funding sources listed in your Funding Information	The authors have declared that no competing interests exist.

later in the submission form are also declared in your Financial Disclosure statement.<br><br>View published research articles from *PLOS ONE* for specific examples.<br><br>**NO authors have competing interests**<br><br>Enter: *The authors have declared that no competing interests exist.*<br><br>**Authors with competing interests**<br><br>Enter competing interest details beginning with this statement:<br><br>*I have read the journal's policy and the authors of this manuscript have the following competing interests: [insert competing interests here]*<br><br>* typeset	
**Ethics Statement**<br><br>Enter an ethics statement for this submission. This statement is required if the study involved:<br><br>• Human participants<br>• Human specimens or tissue<br>• Vertebrate animals or cephalopods<br>• Vertebrate embryos or tissues<br>• Field research<br><br>Write "N/A" if the submission does not require an ethics statement.<br><br>General guidance is provided below. Consult the submission guidelines for detailed instructions. **Make sure that all information entered here is included in the Methods section of the manuscript.**	The collection of plant materials used in our study complied with permission of related institutions, and complied with national or international guidelines and legislation. The experiments did not involve endangered or protected species.

**Format for specific study types**<br><br>**Human Subject Research (involving human participants and/or tissue)**<br>• Give the name of the institutional review board or ethics committee that approved the study<br>• Include the approval number and/or a statement indicating approval of this research<br>• Indicate the form of consent obtained (written/oral) or the reason that consent was not obtained (e.g. the data were analyzed anonymously)<br><br>**Animal Research (involving vertebrate animals, embryos or tissues)**<br>• Provide the name of the Institutional Animal Care and Use Committee (IACUC) or other relevant ethics board that reviewed the study protocol, and indicate whether they approved this research or granted a formal waiver of ethical approval<br>• Include an approval number if one was obtained<br>• If the study involved *non-human primates*, add *additional details* about animal welfare and steps taken to ameliorate suffering<br>• If anesthesia, euthanasia, or any kind of animal sacrifice is part of the study, include briefly which substances and/or methods were applied<br><br>**Field Research**<br><br>Include the following details if this study involves the collection of plant, animal, or other materials from a natural setting:<br>• Field permit number<br>• Name of the institution or relevant body that granted permission	
**Data Availability**<br><br>Authors are required to make all data underlying the findings described fully available, without restriction, and from the time of publication. PLOS allows rare exceptions to address legal and ethical concerns. See the PLOS Data Policy and FAQ for detailed information.	Yes - all data are fully available without restriction

A Data Availability Statement describing where the data can be found is required at submission. Your answers to this question constitute the Data Availability Statement and **will be published in the article**, if accepted.<br><br>**Important:** Stating 'data available on request from the author' is not sufficient. If your data are only available upon request, select 'No' for the first question and explain your exceptional situation in the text box.<br><br>Do the authors confirm that all data underlying the findings described in their manuscript are fully available without restriction?	
**Describe where the data may be found in full sentences. If you are copying our sample text, replace any instances of XXX with the appropriate details.**<br><br>• If the data are **held or will be held in a public repository**, include URLs, accession numbers or DOIs. If this information will only be available after acceptance, indicate this by ticking the box below. For example: *All XXX files are available from the XXX database (accession number(s) XXX, XXX.*).<br>• If the data are all contained **within the manuscript and/or Supporting Information files**, enter the following: *All relevant data are within the manuscript and its Supporting Information files.*<br>• If neither of these applies but you are able to provide **details of access elsewhere**, with or without limitations, please do so. For example:<br><br>*Data cannot be shared publicly because of [XXX]. Data are available from the XXX Institutional Data Access / Ethics Committee (contact via XXX) for researchers who meet the criteria for access to confidential data.*<br><br>*The data underlying the results presented in the study are available from (include the name of the third party*	The data sets supporting the results of this article are included within the article and its additional files. All the raw data of the transcriptome sequence have been deposited in the NCBI BioProject database (https://www.ncbi.nlm.nih.gov/bioproject/PRJNA908908) under the BioProject PRJNA908908.

and contact information or URL).<br>• This text is appropriate if the data are owned by a third party and authors do not have permission to share the data.<br><br>* typeset	
Additional data availability information:	Tick here if the URLs/accession numbers/DOIs will be available only after acceptance of the manuscript for publication so that we can ensure their inclusion before publication.

Powered by Editorial Manager® and ProduXion Manager® from Aries Systems Corporation

Manuscript

Click here to access/download;Manuscript;manuscrip-sunflower Plos one (R2-clean).doc

Comparative Transcriptome Analysis in Two Contrasting Genotypes for *Sclerotinia Sclerotiorum* Resistance in Sunflower

Mingzhu Zhao[1], Bing Yi[1], Xiaohong Liu[1], Dexing Wang[1], Dianxiu Song[1], Enyu Sun[1], Liangji Cui[1], Jingang Liu[1*], Liangshan Feng[2*]

,

[1]Institute of Crop Research, Liaoning Academy of Agricultural Sciences, Shenyang 110161, China

[2] Liaoning Academy of Agricultural Sciences, Shenyang 110000, China

* Corresponding author: Jingang Liu (jingangliu2022@163.com)

Liangshan Feng (fenglsh@163.com)

Abstract

Sclerotinia sclerotiorum as a necrotrophic fungus causes the devastating diseases in many important oilseed crops worldwide. The preferred strategy for controlling S. sclerotiorum is to develop resistant varieties, but the molecular mechanisms underlying *S. sclerotiorum* resistance remain poorly defined in sunflower (*Helianthus annuus*). Here, a comparative transcriptomic analysis was performed in leaves of two contrasting sunflower genotypes, disease susceptible (DS) B728 and disease resistant (DR) C6 after *S. sclerotiorum* inoculation. At 24 h post-inoculation, the DR genotype exhibited no visible growth of the hyphae as well as greater activity of superoxide dismutase activity (SOD), peroxidase (POD), catalase (CAT), glutathione-S-transferase (GST), ascorbate peroxidase (APX) and monodehydroascorbate reductase (MDAR) than DS genotype. A total of 10151 and 7439 differentially expressed genes (DEGs) were detected in DS and DR genotypes, respectively. Most of DEGs were enriched in cell wall organisation, protein kinase activity, hormone, transcription factor activities, redox homeostasis, immune response, and secondary metabolism. Differential expression of genes involved in expansins, pectate lyase activities, ethylene biosynthesis and signaling and antioxidant activity after *S. sclerotiorum* infection could potentially be responsible for the differential resistance among two genotypes. In summary, these finding provide additional insights into the potential molecular mechanisms of *S. sclerotiorum*'s defense response and facilitate the breeding of *Sclerotinia*-resistant sunflower varieties.

Keywords: Differentially expressed genes; Molecular mechanisms; Sunflower (*Helianthus annuus*); *Sclerotinia sclerotiorum*; Transcriptomic

Introduction

Sunfower (*Helianthus annuus*), is the third oilseed crop in the world, primarily for production of high-quality cooking oil and confectionary products and bird food, among others [1]. The occurrence of *Sclerotinia* and the lack of Sclerotinia-resistant varieties led to the decline of the planting area and yield in sunflower. *Sclerotinia sclerotiorum* (Lib.) de Bary is a kind of necrotrophic fungus, which infects over 400 species of plants worldwide including important oilseed crops, such as soybean, canola and sunflower [2-4]. Crop productivity and oil quality can be negatively affected by stem rot, root rot and head rot of plant at different developmental stages [5-6]. Fungal pathogens penetrates the host cuticle first forms an infection pad through mechanical pressure or secreting keratinase, then produce oxalic acid, toxic compounds, cell-wall-degrading enzymes and develop water-soaked lesions, tissue necrosis and finally bear sclerotia [7]. Although a few fungicides are available to manage this disease, the little efficiencies, the environmental costs cannot be ignored [8]. Breeding and cultivating resistant varieties are the best strategy to control *S. sclerotiorum* [9-10]. For the effective breeding of *Sclerotinia*-resistant varieties, therefore, it is essential to understand the genetic and molecular basis of the interactions of *S. sclerotiorum* with its host crops.

Pathogen-associated molecular pattern-triggered immunity (PTI) and effector-triggered immunity (ETI) as the major immune systems in plants have been evolved to cope with pathogens [11]. For pathogen recognition, PTI uses cell-surface pattern recognition receptors (PRRs) that respond to pathogen-associated molecular pattern. The early reactions of PTI include calcium influx, production of reactive oxygen species (ROS), and activation of mitogen activated protein kinases (MAPKs) to trigger transcriptional processes regulated by plant transcription factors [12]. PTI plays an important role in plant resistance to *S. sclerotiorum* [13]. However, adapted pathogens able to suppress PTI may be recognized by plant intracellular resistance proteins of the nucleotide-binding and leucine-rich repeat (NLR) family, encoded by resistance (R) genes, leading to ETI [14, 15]. ETI triggers a series of defense responses in the host plant, most commonly at the infection site causing programmed cell death and anaphylaxis (thereby limiting the growth, reproduction, and expansion of the pathogen), and this is a very effective way for biotrophs fungi, but for necrotrophs fungi, it can kill the host cells and feed on the contents, while biotrophs

complete their life cycle depending on the living host cells [16]. To date, no PRRs or R proteins involved in recognizing *S. sclerotiorum* in host plants have been observed [17, 18].

Transcriptional profling at the site of infection provides an efficient means to reflect the natural epidemiologic cycle among responses between resistant and susceptible varieties [19]. Previous studies characterized the transcriptomic changes in soybean, *Brassica napus*, and *Arabidopsis thaliana* during the defense responses to *S. sclerotiorum*, which involves rapid induction of key pathogen responsive genes, including glucanases, chitinases, peroxidises and WRKY transcription factor family, pathogenesis related proteins, as well as genes related to signal transduction, cellular redox state, cell wall composition and hormone signaling pathways [20, 21]. However, the molecular regulatory mechanisms of sunflower in response to *S. sclerotiorum* remain poorly understood. In this study, we performed a leaf transcriptomic analysis of resistant and susceptible sunflower to determine the defense responses to *S. sclerotiorum* using RNA sequencing (RNA-seq). This work could provide new insight into understanding of the molecular mechanisms underlying the interaction between *S. sclerotiorum* and sunflower.

Materials and methods

Plant materials, inoculation and sampling

Both DR genotype C6 and DS genotype B728 are oilseed sunflower inbred lines which were bred by Liaoning Academy of Agricultural Sciences. The plants of these lines, which had similar growth periods, were cultivated in a growth chamber under a 16 h/8 h light/dark cycle (light intensity 4000 Lux) at 25℃. The infected inflorescences were sampled from field-grown sunflowers in Shenyang, Liaoning Province, China, to isolate and purify the *S. sclerotiorum*. The strain of *S. sclerotiorum* in present study was isolated and maintained on potato dextrose agar medium. A single mycelial plug (3 mm diameter) was cut from the culture to place at the center of a new potato dextrose agar plate for incubation at 25℃ for 5 d in the dark. A total of ten mycelial agar plugs were collected from the edge of the colony for incubation in another new potato dextrose agar plate with the same conditions. Mycelial agar plugs were excised from the margin of a growing colony and used to inoculate sunflower leaves of 6 week old, while potato dextrose agar

agar plugs without fungus was used as a mock-inoculated control. A randomized complete-block was used in the experimental design with three replications, and each replicate contained 20 plants. As the visible lesions appeared in DS genotype rather than DR genotype at 24 h post-inoculation (hpi), then the 20 leaves samples within the range of 0.5 cm bordering the extending lesion from inoculated and mock-inoculated plants were harvested for each replicate. Immediately after sampling, all samples were washed with sterilized water and placed into storage tubes, frozen with liquid nitrogen, and then stored at -80℃ for biochemical assays and RNA isolation.

Microscopic observation

To observe the growth of *S. sclerotiorum* in sunflower, the leaves samples from inoculated plants of both genotypes were sampled at 24 hpi for wheat germ agglutinin (WGA) staining [22]. Single thin shavings were cut from the leaves samples, and placed in 2 ml microcentrifuge tubes. Then 10% KOH was used to soake the samples at room temperature, which had been rinsed once with distilled water. After rinsing three times with distilled water later, the samples were incubated with WGA stain solution, which ~~was consisting~~ of 10 μg/ml WGA, 0.02% (v/v) Tween 20, 10 mM phosphate-buffered saline, pH 7.4. By using vacuum infiltration for 30 min, the samples were ~~washing~~ three times with phosphate-buffered ~~saline. Then, we finally~~ observed ~~the samples~~ under a FV 1000 fluorescence microscope (OLYMPUS) with excitation at 488 nm and emission at 500 to 540 nm.

Antioxidant enzyme extraction and activity assays

To obtain the enzymatic extract, frozen leaves (500 mg) from each sample was ground in liquid nitrogen to a fine powder. The tissue ~~were grinded with borate buffer, and then the homogenate volume reached a final volume of 10 mL with borate buffer~~ and centrifuged at 10,000 rpm for 15 min at 4℃.

The superoxide dismutase activity (SOD) activity was determined as of the ability of photochemical reduction of nitrotetrazolium according to the method described by Giannopolitis and Ries [23]. The peroxidase (POD) activity was carried out by the method of Shannon et al [24]. The activity of catalase (CAT) was determined according to the method of Aebi [25]. The

glutathione-S-transferase (GST) activity was measured as described by Mauch and Dudler [26]. The ascorbate peroxidase (APX) activity was obtained according to Nakano and Asada [27], using ascorbic acid as a substrate. The monodehydroascorbate reductase (MDAR) activity was assayed according to Drazkiewicz et al [28]. All the enzymatic analyses were performed in triplicate.

RNA sequencing and analysis of RNA-seq data

Sunflower leaves were extracted using the RNAprep Pure Plant Kit (TIANGEN Biotech, Beijing, China) according to the instructions provided by the manufacturer. RNA samples were used to measure the concentration and purity in NanoDrop 2000 (Thermo Fisher Scientific, Wilmington, DE). The integrity of RNA samples was measured by using the RNA Nano 6000 Assay Kit of the Agilent Bioanalyzer 2100 system (Agilent Technologies, CA, USA). In this study, we used RNA samples with a 260/280 ratio of 1.96-2.17, a 28S/18S ratio of 1.0, and a RNA integrity number greater than 8.0 to prepare RNA-Seq libraries using NEBNext UltraTM RNA Library Prep Kit (NEB, USA) according to the manufacturer’s instructions. The clustering of the index-coded samples was performed on a cBot Cluster Generation System using TruSeq PE Cluster Kit v4-cBot-HS (Illumia). Finally, the RNA sequencing was performed on IlluminahpiiSeq 2500 platform by the Biomarker Technology, Qingdao, China.

Sequencing data were analyzed with the help of a bioinformatic pipeline tool, the BMKCloud platform (www.biocloud.net). Raw reads of fastq format were firstly processed through in-house perl scripts. In this step, the clean reads were obtained by removing adapter-containing reads, ploy-N-containing reads, and low quality reads from raw data. The Q20, Q30, GC-content and sequence duplication level of the clean data were calculated. All the downstream analyses were based on clean data with high quality. The adaptor sequences and low-quality sequence reads were removed from the data sets. Raw sequences were transformed into clean reads after data processing. These clean reads were then mapped to the reference genome sequence. Only reads with a perfect match or one mismatch were further analyzed and annotated based on the sunflower reference genome. The HanXRQr1.0 genome was used as reference in the present study [29]. Then, Hisat2 tools soft were used to map with reference genome. The StringTie Reference Annotation Based Transcript (RABT) assembly method was used to construct and identify both

known and novel transcripts from Hisat2 alignment results. The gene expression levels are reflected by fragments per kilobase of transcript per million fragments mapped (FPKM). Genes with low expression (FPKM <1) ~~will be~~ eliminated by default in the analysis process. An analysis of differential expression was carried out using the DESeq2 package. The false discovery rate (FDR) was controlled by using the Benjamini and Hochberg's approach to adjust the resulting P values. The genes were identified as DEGs when the FDR ≦ 0.05 as well as the fold change ≧ |2|.

Functional classification and pathway analysis

Enrichment of Gene Ontology (GO) terms of the DEGs was implemented by AgriGO v2.0 (http://systemsbiology.cau.edu.cn/agri-GOv2/) with hypergeometric statistical test and Hocberg (FDR). The Kyoto Encyclopedia of Genes and Genome (KEGG, http://www.genome.jp/kegg/) was utilized for pathway enrichment analysis of DEGs. The overview of metabolic pathways in which DEGs involved in response to *S. sclerotiorum* were visualized by using MapMan software. In the MapMan software, the natural log of the ratio of the experimental mean to the control mean of the above called DEGs was taken in input files. Final analyses were performed with MapMan version 3.6.0 including the automatic application of the Wilcoxon rank sum test. Mapping files were exported from MapMan.

Validation of RNA-seq analysis by qPCR

The extracted RNA was also used for qRT-PCR analysis to confirm the DEG results from RNA-seq. The same samples for RNA-seq were also used to extract the total RNA, which was further used to synthesize the cDNA. The qRT-PCR was conducted using ChamQ Universal SYBR qPCR Master Mix (Vazyme Biotech co., ltd, NanJing, China) on a Light Cycler 480 II (Roche, Basel, Switzerland). Primer sequences are described in S1 Table. The cycle threshold (CT) values obtained from three replicates and four technical replicates. The fold changes were calculated by $2^{-\Delta\Delta CT}$. $\Delta\Delta CT = (CT_{target}-CT_{action})_{treatment} - (CT_{target}-CT_{action})_{control}$. Additionally, the results of qRT-PCR were presented as log_2 fold changes to assess the accuracy of the RNA-seq.

Results

Symptoms and physiological traits in leaves of sunflower inoculated with *S. sclerotiorum*

Leaves from 6-week-old plants of two sunflower inbred lines, DR genotype C6 and DS genotype B728, were inoculated with isolated *S. sclerotiorum* using mycelial agar plugs. Visible lesions appeared in DS genotype at 24 hpi (Fig. 1A) and in DR genotype at approximately 72 hpi (Fig. 1C). The lesions developed more rapidly in DS genotype than in DR genotype. After 48 hpi (Fig. 1B&C), the lesions on the leaves of DR genotype were significantly smaller than those on those of DS genotype. By electron microscopy, we found chlorosis ~~phenomenon~~ in the leaf tissue of DS genotype (Fig. 1D) ~~rather than~~ DR genotype at 24 hpi (Fig. 1E). Microscopic assessments of the hyphae further showed that the pathogen was well established in the host tissue and led to extensive mycelial growth in DS genotype at 24 hpi (Fig. 1F), while no visible growth of the hyphae was found in DR genotype (Fig. 1G).

The physiological response of leaves to *S. sclerotiorum* inoculation was further evaluated based on the activity of antioxidant enzymes, such as SOD, POD, CAT, GST, APX and MDAR. These data are shown in Table 1. Following infection, the activity of POD, CAT and GST were increased significantly in both genotype, while the SOD activity was increased significantly in DR genotype rather than DS genotype. APX and MDAR activity in DS genotype was decreased significantly by *S. sclerotiorum* infection, while DR genotype showed non-significant change. *S. sclerotiorum* infection, caused higher activity of the enzymes relative to the DS genotype.

RNA-seq analysis and identification of DEGs

~~By RNA-seq,~~ average of 43 million raw reads were obtained for each sample, and 74.8-91.8% of trimmed reads were mapped to the reference genome. With a cutoff value of 1, a total of 22027 and 22579 genes were expressed in mock-inoculated samples of DS and DR genotypes, respectively, while 20711 and 23636 in the *S. sclerotiorum*-inoculated samples. The DR genotype constitutively expressed more genes than did the DS genotype.

In order to determine whether DS and DR genotypes respond differently to *S. sclerotiorum* inoculation, DEGs were obtained by comparing gene expression levels between *S. sclerotiorum*- and mock-inoculated samples of the same genotype. In DS genotype, 10151 DEGs were identified between *S. sclerotiorum*- and mock-inoculated samples: 5481 up-regulated and 4670 down-regulated, whereas the same analysis in the DR genotype revealed only 7439 DEGs: 4221 up-regulated and 3218 down-regulated (Fig. 2A). Venn diagrams showed that 2260 and 1000 genes were uniquely up-regulated in DS and DR genotypes, respectively, while 2557 and 1105 were uniquely down-regulated, respectively (Fig. 2B). In addition, 119 genes were only induced in DS genotype after *S. sclerotiorum* infection but have constitutively expressed in the DR genotype (S2 Table).

Gene ontology (GO) enrichment analysis of DEGs

AgriGO software was used to calculate the singular enrichment analysis (SEA) for the up- and down-regulated genes in DS genotypes, highlighted 126 and 134 GO significantly enriched terms, respectively (S3-4 Table), while 98 and 88 GO terms were significantly enriched the up- and down-regulated genes in DR genotype (S5-6 Table). We identified 249 and 282 common GO terms that were enriched in both the genotypes from up- and down-regulated genes in response to *S. sclerotiorum*, respectively (S7-8 Table), which play an important role in the disease resistance of sunflower. For the up-regulated genes, 155 GO terms including protein kinase activity, protein autophosphorylation, response to oxidative stress, chitin catabolic process, immune response, cell surface receptor signaling pathway, nucleotide binding, plasma membrane and peroxidase activity were more enriched in DR genotype than in DS genotype (S7 Table). Among the 145 GO terms for the down-regulated genes, the more enriched terms in DS genotype than in DR genotype included photorespiration, chlorophyll biosynthetic process, photosynthesis, chloroplast, plastid, thylakoid, organelle subcompartment, oxidoreductase activity and chlorophyll binding (S8 Table).

The SEA analysis was carried out on the uniquely up- and down-regulated genes in DS genotypes. Among the uniquely up-regulated genes 26 GO terms were significantly enriched: “intracellular protein transport” in the BP category, “clathrin vesicle coat” in CC category, and “GTPase activity” in MF category (S9 Table). “Carbohydrate metabolic process” in BP category,

“vacuole” in CC category and “metal ion binding” in MF category were the most enriched GO terms among the 118 identified from unique down-regulated genes in the DS genotype (S10 Table).

The SEA analysis was also carried out on the uniquely up- and down-regulated genes in DR genotypes. Among the uniquely up-regulated genes 63 GO terms were significantly enriched: “cellular polysaccharide metabolic process” in the BP category, “cellular polysaccharide biosynthetic process” in CC category, and “cell morphogenesis” in MF category (S11 Table). “Cellular component organization” in BP category, “plasma membrane” in CC category and “transcription factor activity, sequence-specific DNA binding” in MF category were the most enriched GO terms among the 16 identified from unique down-regulated genes in the DR genotype (S12 Table).

Top twenty uniquely up- ($log_2FC > 7$) or down-regulated ($log_2FC < -4$) genes were identified in the DR genotypes, respectively (Table 2 and 3), most of which were enriched in the above uniquely up- or down-regulated GO terms. These uniquely up-regulated genes in the DR genotype were annotated as cytokinin dehydrogenase, cytochrome P450, expansin, transcription factor ERF, TMV resistance protein, etc (Table 2). These uniquely down-regulated genes in DS genotype were annotated as proline-rich protein, dehydration-responsive element-binding protein, leucine-rich repeat receptor protein kinase, bidirectional sugar transporter SWEET14, *etc* (Table 3).

Pathway analysis of DEGs

KEGG pathway analysis was performed on the DEGs in response to *S. sclerotiorum* infection in DS (Fig. S1) and DR (Fig. S2) genotypes. The plant-pathogen interaction was the top up-regulated pathway for both genotypes. In this pathway, 24 and 23 receptor-like proteins, which could be considered as PRRs, were up-regulated significantly by *S. sclerotiorum* infection in DS and DR genotypes, respectively (Table 4). Of these, 13 were uniquely up-regulated in DR genotype and its expression exhibited non-significantly changed in DS genotype, such as EIX1 and EIX2 homolog genes.

By using MapMan software, a pathway was shown ~~which~~ consisting of 1089 and 886 DEGs by *S. sclerotiorum* infection in DS and DR genotypes (Fig. 3). In both genotypes, most of genes

involved in effector receptor (NLR), protein kinase MAPK, calcium-dependent signaling (*e.g.* CDPKs, CBLs and CMLs), transcription factor (*e.g.* ERF, WRKY and MYB), systemic acquired resistance (*e.g.* regulatory protein CBP60/SARD, disease resistance mediator MLO2/6/12, aminotransferase ALD1, pipecolate N-hydroxylase FMO1 and pipecolate oxidase SOX) and secondary metabolism (*e.g.* mevalonate pathway, methylerythritol phosphate pathway, p-coumaroyl-CoA biosynthesis and flavonoid biosynthesis) were up-regulated, ~~whilst most of genes for~~ proteolysis (*e.g.* cysteine-type peptidase, serine-type peptidase, aspartic-type peptidase and metallopeptidase activities) were down-regulated by *S. sclerotiorum* infection. Of above pathways, the number of up- or down-regulated genes in DS genotype was more than in DR genotype. However, a large number DEGs were also divided into hormone signaling, cell wall organisation and redox homeostasis~~, which were chosen to conduct a deeper~~ evaluation of *S. sclerotiorum* resistant traits.

Plant hormone signaling pathways

~~By *S. sclerotiorum* infection, most of genes related to~~ auxin signaling pathways were significantly down-regulated~~, and the number of~~ these down-regulated genes were more in DS genotype than in DR genotype (S13 Table). However, most of genes related to signaling pathways of ethene (ET) and brassinolide (BR), were significantly up-regulated. The DS genotype had more up-regulated genes than the DR genotype in above pathways apart from ET signaling. The ET signaling pathways showed more up-regulated genes in DR genotype (11) compared to DS genotype (7), which was attributed to more uniquely up-regulated genes for receptor protein (ETR/ERS) and EIN3 transcription factor activity (Fig. 4A). After *S. sclerotiorum* infection, only the gene LOC110884479 encoding EIN3 transcription factor activity showed higher expression level in DR genotype than in DS genotype.

Redox homeostasis

Redox homeostasis was affected by *S. sclerotiorum* infection, in which 94 DEGs in DS genotype and 68 in DR genotype. Most of genes for NADPH respiratory burst oxidase homolog involved in the ROS generation were up-regulated in both genotypes (S14 Table). In ROS scavenging, the DR genotype showed more up-regulated genes associated with SODs than the DS genotype (Fig. 4B). On the contrary, the DS genotype had more down-regulated genes involved in ascorbate-based redox regulation including APX and MDAR than the DR genotype. After *S. sclerotiorum* infection,

the DR genotype showed six up-regulated and four down-regulated genes as compared to DS genotype.

Cell wall organisation

In cell wall organisation, 285 and 262 DEGs were identified by *S. sclerotiorum* infection in DS and DR lines, respectively. Most of genes involved in the cellulose synthase complex, xylan biosynthesis, heteromannan biosynthesis, and cutin polyester biosynthesis were down-regulated in both genotypes, while monolignol biosynthesis and expansin activities were up-regulated (S15 Table). However, we found several up-regulated genes related to expansin activities, such as alpha-class expansin, in which more in DR genotype compared to DS genotype (Fig. 4C). In addition, genes involved in pectin modification and degradation, such as pectate lyase showed a peculiar behaviour, most of them were up-regulated in DR genotype rather than DS genotype (Fig. 4E). After *S. sclerotiorum* infection, four genes LOC110871669, LOC110873721, LOC110904631 and LOC110941814 encoding pectate lyase showed higher expression level in DR genotype than in DS genotype.

Validating the reliability of RNA-seq data by qRT-PCR

Nine genes were selected to validate the reliability of RNA-seq data by using qRT-PCR. The expression trends of selected genes obtained by qRT-PCR were good agreement with those measured by RNA-seq (Fig. 5). Of these, the CDPK9 (LOC110873588) and MAPK5 (LOC110935435) were significantly up-regulated by *S. sclerotiorum* infection in both genotypes (Fig. 5A and B). The CML23 (LOC110869082) and five NLRs (LOC110915177, LOC110877402, LOC110923780, LOC110883529 and LOC110929730) were uniquely up-regulated in the DR genotype and their expression but showed non-significantly changed in DS genotype (Fig. 5C-H). Notably, the LOC110935776 encoding NLR exhibited a the opposite expression pattern, which was down-regulated in DS genotype but dramatically up-regulated in the DR genotype (Fig. 5I).

Discussion

Differences in *S. sclerotiorum* resistance between the two sunflower genotypes

In this study, the DR genotype C6 displayed symptoms of *S. sclerotiorum* infection later (72 hpi) and with less severity after inoculation than DS genotype B728 (24 hpi), which was also reported by previous studies in regard to the defence response to *S. sclerotiorum* in other crop plants [30,31]. The assessments of visible lesions and hyphae further demonstrated the resistance difference between these two genotypes. We sampled the inoculated and mock-inoculated leaves at 24 hpi for RNA-seq analysis, when the slight phenotypic difference has just occurred. At this time point, the DS genotype had larger number of DEGs than the DR genotype, indicating that it is relatively hyper-responsive or more sensitive to *S. sclerotiorum* at the molecular level. The results were inconsistent with the results of previous studies in B. *napus*[30,32].

Expansin and pectate lyase activities contribute to resistance against *S. sclerotiorum*

We identified more down-regulated genes involved in chloroplast as well as photosynthesis in DS genotype compared with those in the DR genotype. Disruption of chloroplast function often resulted in the differentially expressed of genes involved in cell wall synthesis and modification. Cell wall, as the first defensive barrier against pathogens, undergoes dynamic remodeling to prevent infection by pathogens [33]. During the interaction with its host, *S. sclerotiorum* synthesizes and secretes oxalic acid and cell-wall-degrading enzymes, which could decrease cell wall rigidity, cause rapid cell collapse, and finally destroy expanding parts of infected tissue [34]. The extracellular enzymes produced by *S. sclerotiorum* can physically degrade plant cell wall components and consequently provide nutrients to drive the infection process [35]. Up-regulation of cellulose synthases, xyloglucan endotransglucosylase/hydrolase, monolignol biosynthesis, lignin deposition could reinforce the cell wall to react faster and stronger to subsequent *S. sclerotiorum* attack [36-38]. In this study, most of genes related to monolignol biosynthesis and expansin activities were up-regulated by *S. sclerotiorum* infection in both genotypes. Importantly, we found more up-regulated genes related to expansin activities in DR genotype compared to DS

genotype. Plant expansins as cell wall protein play a key role in many processes modify the cell wall, including relaxation and extension [39], and constitute a regular component against pathogen infection [40]. Overexpression of *EXLB8*, one of subfamilies of plant expansins, improved resistance to *S. sclerotiorum* in tobacco through physically modifying the cell wall structure as well as activating the phytohormone signaling pathways, such as JA-ET cooperation [41].

In addition, we found that the genes for pectate lyase were up-regulated in DR genotype rather than DS genotype by *S. sclerotiorum* infection. The homogalacturonan of pectin that could expose the polysaccharides at the early stage of invasion, was depolymerized by pectate lyase, which was released from pathogens [42-44]. By the degradation of polysaccharides, the plant cell wall produce oligosaccharides, which can enhance disease resistance via promoting ROS burst, MAPK activation and thus the expression of immune-responsive genes[45,46]. Our results suggested that the expansin and pectate lyase activities may be associated with *Sclerotinia* resistance in sunflower.

Differences in ROS scavenging between contrasting genotypes

The ROS are secondary messengers in the signal transduction process and play crucial roles in stress response and plant growth [47]. However, if the stress is too severe, excess ROS will cause programmed cell death and trigger host cell necrosis, which can promote the growth of necrotrophic pathogens such as *S. sclerotiorum* [48]. Organisms have complex antioxidant systems that regulate the balance between ROS production and ROS scavenging to combat oxidative stress caused by excess ROS [49]. SOD is involved in inactivating superoxide anions, which constitutes the front-line defense against ROS [50]. In this study, the increase of SOD activity was found only in the DR genotype after *S. sclerotiorum* infection. Most of genes associated with SOD were up-regulated in the DR genotype, indicated an elevated levels of antioxidant defence decreased oxidative damage and cell death in this genotype. Moreover, the pathway of ascorbate-glutathione cycle is essential for ROS scavenging, which can protect ROS signalling and avoid H_2O_2 toxicity [51,52]. We found higher activity of GST, APX and MDAR in DR genotype than in DS genotype after *S. sclerotiorum* infection. GSTs play a crucial role as cellular protectants in detoxification against oxidative damage, and were induced by *S.*

sclerotiorum in this study, which has been also reported in the previous studies [53]. However, the DS genotype had more down-regulated genes associated with APX and MDAR involved in ascorbate-based redox regulation. APX as one of the most important peroxidases catalyzes reduction of H_2O_2 by the reducing power of ascorbate [54], which is the most important reducing substrate for H_2O_2 detoxification in plant cells [55]. MDAR can directly catalyses the regeneration of ascorbate at both plasmalemma and thylakoid membrane [56]. During the detoxification of ROS, Additionally, MDAR is capable of converting phenoxyl radicals into their respective parental phenols, which are very efficient antioxidants comparable to ascorbate [57]. Decreased activity of APX and MDAR only in DS leaves likely contributes to the cellular degradation before fungal hyphae, thereby increasing nutrient availability after OA-induced cell death [58].

Immune response to *S. sclerotiorum* in sunflower

The initial stage of PTI involves PRR stimulation [58]. A total of 57 genes for receptor-like kinases (RLKs) were as well-characterized PRR, which were up-regulated in *Brassica napus* by *S. sclerotiorum* infection [30]. A PRR gene, *ZmWAK*, was identified in maize by QTL mapping, which was up-regulated after infected and confered quantitative resistance to head smut [57]. In this study, 24 and 23 genes, in the DR and DS genotype, respectively, were identified as PRRs in the plant-pathogen interaction pathway, which were up-regulated by *S. sclerotiorum* infection. Importantly, we found 13 PRRs were uniquely up-regulated in the DR genotype, which might have a critical role in recognizing pathogen-associated molecular patterns induced by S. sclerotiorum and initiating the immune response.

Defense responses also begin with the intracellular recognition of the pathogen effector molecules through a series of plant R gene products, which leads to ETI [58]. In disease resistance breeding, ETI as an important trait is widely used to resist the biotrophic and hemibiotrophic instead of necrotrophic pathogens [59]. However, in *Arabidopsis*, RLM3 has been identified as R gene, which encodes a TIR domain known to affect broad-range immunity to several necrotrophic pathogens [60]. It is unknown whether sunflower has any R-genes may refered to the *S. sclerotiorum* resistance. In this study, we identified six R genes encoding NLR proteins by qRT-PCR, which were uniquely up-regulated in the DR genotype. Of these, the gene

LOC110935776 was down-regulated in DS genotype but dramatically up-regulated in DR genotype. In sunflower, it will be necessary to investigate whether these NLR genes play a role in the immune response to *S. sclerotiorum*.

Defense signaling pathways in response to *S. sclerotiorum* challenge

When recognizing invading pathogens, plants respond to pathogen attack by activation a large number of defense responses, in which a central role is signaling, including MAPK kinases, calcium-dependent signaling, plant hormone and transcription factors. Most of genes associated with MAPK kinases, CDPKs, CMLs, ET, BR, ERF, WRKY and MYB were up-regulated by *S. sclerotiorum* infection in this study. During *S. sclerotiorum* infection, MAPK is an important serine/threonine protein kinase that transduces signals from cytoplasm to nucleus [61]. The changes of transcription factors dependent on a series of phosphorylation was due to the MAPK cascade signalling pathway, which were activated after PRR stimulation [62,63]. The transcription factors MPK4 and the WRKY33 were demonstrated to assoiated with the resistance to *Botrytis cinerea* and *S. sclerotiorum* [64-66]. Over-expression of BnMAPK4 enhances resistance to *S. sclerotiorum* in transgenic *Brassica napus* [67].

In addition, the plant respond to *S. sclerotiorum* attack was also depend on the calcium (Ca) signaling pathway, which mainly included CDPKs and CMLs. It is ~~a~~ well known that the H_2O_2 accumulation can be positively regulated by CDPKs via phosphorylating in the plasma membrane and cytosol, and thus activate RBOHD/F [68]. These results suggest that these genes as the regulator might play a key role in cellular signaling networks to induce *S. sclerotinia* stress resistance. In this study, one gene CML23 were identified as uniquely up-regulation in the DR rather than in DS genotype.

Jasmonic acid (JA)/ET and SA-mediated signaling pathways are known to be major components of plant defense networks [69,70], which can act independently, synergistically, or in some cases antagonistically between host/pathogens [71]. In this study, genes associated with ET signal pathways were induced, whilst no salicylic acid (SA) and JA responsive genes were identified. ET could induce the pathogenesis related proteins and synthesize the phytoalexin against invading microbial pathogens [72]. Previous study showed that the ET responsive

signaling seemed to proceed at a late stage of disease in the tolerant *B. carinata* but no such increase was found in the susceptible ones at any of the investigated time points [73]. We also found that the DR genotype had more up-regulated genes associated with ETR/ERS and transcription factor EIN3 in the ET signaling pathways compared to DS genotype. Our results suggested that the increase of ET signal transduction could contribute to resistance against *S. sclerotiorum*. Oppositely, more up-regulated genes for ET biosynthesis were found in DS genotype than in DR genotype (S13 Table). ET has been demonstrated to play a key role in the programmed cell death regulation [74], which might help the necrotrophic pathogen to absorb nutrients for continued growth and development [70]. Thus, we proposed that blocking ET biosynthesis may alleviate *S. sclerotinia* in sunflower.

Conclusions

RNA-Seq analysis was carried ~~out in this study~~ to analyze transcriptomic profiles of two sunflower genotypes viz., DS and DR that were systemically infected with *S. sclerotinia* and elucidate the molecular basis of the *S. sclerotiorum* resistance mechanism. The DEGs analysis showed that the DR genotype is less affected by *S. sclerotiorum* infection as compared to the DS genotype. Taken together the results from the GO, KEGG and MapMan analysis, a model was proposed to show the major molecular mechanisms underlying the defense responses against *S. sclerotiorum* in sunflower (Fig. 6). The oxalic acid and cell-wall-degrading enzymes as well as the excess of ROS, generated by the interaction between host and *S. sclerotiorum*, cause the programmed cell death, which help the *S. sclerotiorum* to obtain nutrients for growth and development. In the DR genotype, the expansins were up-regulated to restrain cell-wall-degrading enzymes; pectate lyase activities were up-regulated to activate the defense response; the SOD was up-regulated to eliminate of ROS; the ET signaling were up-regulated to activate the systemic acquired resistance and secondary metabolisms via a series of signal transduction. In DS genotype, however, the up-regulated of ET biosynthesis and the down-regulated of APX and MDAR facilitated the programmed cell death. Our results revealed how the cell wall modifying enzymes, antioxidants, hormone as well as signaling increased *S. sclerotiorum* resistance in DR relative to DS sunflower genotype. Overall, these finding will serve as a resource for further studies of the molecular mechanisms associated the *S. sclerotiorum* resistance in sunflower and development of

effective strategies in *Sclerotinia*-resistance breeding.

Availability of data and materials

The data sets supporting the results of this article are included within the article and its additional files. All the raw data of the transcriptome sequence have been deposited in the NCBI BioProject database (https://www.ncbi.nlm.nih.gov/bioproject/PRJNA908908) under the BioProject PRJNA908908.

Author Contributions

Funding acquisition: Mingzhu Zhao, Liangji Cui, Bing Yi.

Methodology: Mingzhu Zhao, Bing Yi

Investigation: Mingzhu Zhao, Xiaohong Liu, Dexing Wang, Dianxiu Song, Enyu Sun.

Supervision: Jingang Liu, Liangshan Feng

Writing-original draft: Mingzhu Zhao.

Writing-review & editing: Jingang Liu, Liangshan Feng

Competing interests

We hereby confirm that all authors have declared that no competing interests exist.

Acknowledgment

We thank Prof. Wenjing Zheng, Institute of Rice Research, Liaoning Academy of Agricultural Sciences, to review the manuscript.

Figure Captions

Fig. 1 Visible lesions of leaves at 24 hpi (A), 48 hpi (B) and 72 hpi (C) and chlorosis phenomenon (D and E) and mycelial growth (F and G) of two genotypes at 24 hpi by electron microscopy. Fungal hyphae shows green fluorescence, cell walls of plant show red fluorescence (F and G) of two genotypes at 24 hpi by Leaf Fungal Hyphae WGA-PI staining.

Fig. 2 Differentially expressed genes (DEGs) in each pairwise comparison. (A) Volcano plot. The x-axis and y-axis represents the log2 (fold change) and −log10 (FDR-value). The red dots represent up-regulated genes with $\log_2$ Fold_change >1 and FDR <0.01 and the green dots represent down-regulated genes with $\log_2$ Fold_change < −1 and FDR <0.01. (B) Based on the two comparisons performed, two Venn diagrams showed the numbers of genes that are up- and

down-regulated. Overlapping circles represent genes up- or down-regulated in both comparisons.

Fig. 3 Infection with *S. sclerotiorum* results in DEGs at the DS (A) and DR (B) genotypes as visualized in MapMan. Red and green indicate DEGs that are up-regulated and down-regulated, respectively.

Fig. 4 Heat map of DEGs related to ET signaling (A), ROS (B), expansin (C) and pectin (D). The gene expression levels were compared between *S. sclerotiorum*- and mock-inoculated samples of the same genotype as well as between the DR and DS genotype under the same conditions.

Fig. 5 The expression pattern of nine genes were verified by qRT-PCR.

Fig. 6 Sunflower defense responses against *S. sclerotiorum* described in a molecular model. CWDE: cell wall-degrading enzymes; OA: oxalic acid; TF: transcription factor; MAPK- mitogen activated protein kinase.

Supporting Information

S1 Table. The gene-specific primers for qRT-PCR.

S2 Table. 119 constitutive expressed genes are induced by *S. sclerotiorum* infection in the DS genotype and are already expressed in the DR genotype in mock-inoculated control.

S3 Table. GO analysis to identify enriched gene ontologies among the up-regulated DEGs in DS genotype B728.

S4 Table. GO analysis to identify enriched gene ontologies among the down-regulated DEGs in DS genotype B728.

S5 Table. GO analysis to identify enriched gene ontologies among the up-regulated DEGs in DR genotype C6.

S6 Table. GO analysis to identify enriched gene ontologies among the down-regulated DEGs in DR genotype C6.

S7 Table. GO analysis to identify enriched gene ontologies among the common up-regulated DEGs in DS and DR genotypes.

S8 Table. GO analysis to identify enriched gene ontologies among the common up-regulated DEGs in DS and DR genotypes.

S9 Table. GO analysis to identify enriched gene ontologies among the unique up-regulated DEGs in DS genotype B728.

S10 Table. GO analysis to identify enriched gene ontologies among the unique down-regulated DEGs in DS genotype B728.

S11 Table. GO analysis to identify enriched gene ontologies among the unique up-regulated DEGs in DT genotype C6.

S12 Table. GO analysis to identify enriched gene ontologies among the unique down-regulated DEGs in DT genotype C6.

S13 Table. DEGs involved in phytohormone action by MapMan analysis.

S14 Table. DEGs involved in redox homeostasis by MapMan analysis.

S15 Table. DEGs involved in cell wall organisation by MapMan analysis.

S1 Fig. Kyoto Encyclopedia of Genes and Genome (KEGG) analysis for up- (A) and down-regulated (B) DEGs in DS genotype, respectively.

S2 Fig. Kyoto Encyclopedia of Genes and Genome (KEGG) analysis for up- (A) and down-regulated (B) DEGs in DR genotype, respectively.

REFERENCES

1.Liu J, Zhang Y, Meng Q, Shi F, Ma L, Li Y. (2017) Physiological and biochemical responses in sunflower leaves infected by *Sclerotinia sclerotiorum*. Physiol Mol Plant P 100:41-48.

2.Vuong TD, Hoffman DD, Diers BW, Miller JF, Steadman JR, Hartman GL. (2004) Evaluation of soybean, dry bean, and sunflower for resistance to *Sclerotinia sclerotiorum*. Crop Sci. 44(3): 777-783.

3.Joshi RK, Megha S, Basu U, Rahman MH, Kav NN. (2016) Genome wide identification and functional prediction of long non-coding RNAs responsive to *Sclerotinia sclerotiorum* infection in *Brassica napus*. PLoS ONE 11: e0158784

4.Prats E, Galindo JC, Bazzalo ME, León A, Macías FA, Rubiales D, et al. (2007) Antifungal activity of a new phenolic compound from capitulum of a head rot-resistant sunflower genotype. J Chem Ecol. 33: 2245-2253.

5.Lu G. (2003) Engineering *Sclerotinia sclerotiorum* resistance in oilseed crops. Arf J Biotechnol. 2:509-516.

6.Clarkson JP, Fawcett L, Anthony SG, Young C. (2014) A model for *Sclerotinia sclerotiorum* infection and disease development in lettuce, based on the effects of temperature, relative humidity and ascospore density. PLoS ONE 9: e94049.

7.Bolton MD, Thomma BP, Nelson BD. (2006) *Sclerotinia sclerotiorum* (Lib.) de Bary: biology and molecular traits of a cosmopolitan pathogen. Mol Plant Pathol 7, 1-16.

8.del Río LE, Bradley CA, Henson RA, Endres GJ, Hanson BK, McKay K, et al. (2007) Impact of *Sclerotinia* stem rot on yield of canola. Plant Dis 91: 191-194.

9.Zhao J, Peltier AJ, Meng J, Osborn TC, Grau CR. (2004) Evaluation of *Sclerotinia* stem rot resistance in oilseed *Brassica napus* using a petiole inoculation technique under greenhouse conditions. Plant Dis 88:1033-1039.

10.Bastien M, Sonah H, Belzile F. (2014) Genome wide association mapping of *Sclerotinia sclerotiorum* resistance in soybean with a genotyping-by-sequencing approach. Plant Genome-US 7:2013-10.

11.Jones JD, Dang JL. (2006) The plant immune system. Nature 444:323-329.

12.Tena G. (2021) PTI and ETI are one. Nat. Plants 7:1527-1527.

13.Gao X, Dang X, Yan F, Li Y, Xu J, Tian S, et al. (2022) ANGUSTIFOLIA negatively regulates resistance to *Sclerotinia sclerotiorum* via modulation of PTI and JA signalling pathways in *Arabidopsis thaliana*. Mol Plant Pathol 23:1091-1106.

14.Chisholm ST, Coaker G, Day B, Staskawicz BJ. (2006) Host-microbe interactions: shaping the evolution of the plant immune response. Cell 124: 803-814.

15.Badet T, Léger O, Barascud M, Voisin D, Sadon P, Vincent R, et al. (2019) Expression polymorphism at the ARPC 4 locus links the actin cytoskeleton with quantitative disease resistance to *Sclerotinia sclerotiorum* in *Arabidopsis thaliana*. New Phytol 222, 480-496.

16.Wen L. (2013) Cell death in plant immune response to necrotrophs. J Plant Biochem Physiol 1: 1-3.

17.Wang JQ, Mao L. (2019) The ERK pathway: molecular mechanisms and treatment of depression. Mol Neurobiol 56:6197-6205.
18.Yang G, Tang L, Gong Y, Xie J, Fu Y, Jiang D, et al. (2018) A cerato-platanin protein SsCP1 targets plant PR1 and contributes to virulence of *Sclerotinia sclerotiorum*. New Phytol 217:739-755.
19.Fass MI, Rivarola M, Ehrenbolger GF, Maringolo CA, Montecchia JF, Quiroz F, et al. (2020) Exploring sunflower responses to *Sclerotinia* head rot at early stages of infection using RNA-seq analysis. Sci Rep-UK 10:1-14.
20.Joshi RK, Megha S, Rahman MH, Basu U, Kav NN. (2016) A global study of transcriptome dynamics in canola (*Brassica napus* L.) responsive to *Sclerotinia sclerotiorum* infection using RNA-Seq Gene 590: 57-67.
21.Girard IJ, Tong C, Becker MG, Mao X, Huang J, de Kievit T, et al. (2017) RNA sequencing of *Brassica napus* reveals cellular redox control of *Sclerotinia* infection. J Exp Bot 68:5079-5091.
22.Ghareeb H, Becker A, Iven T, Feussner I, Schirawski J. (2011) Sporisorium reilianum infection changes inflorescence and branching architectures of maize. Plant Physiol 156: 2037-2052.
23.Giannopolitis CN, Ries SK. (1977) Superoxide dismutases: II. Purification and quantitative relationship with water-soluble protein in seedlings. Plant Physiol 59(2):315-318.
24.Shannon LM, Kay E, Lew JY. (1996) Peroxidase isozymes from horseradish roots I. Isolation and physical properties. J Biol Chem 241:2166–2172.
25.Aebi H. (1984) Catalase in vitro. Method Enzymol 105:121–126.
26.Mauch F, Dudler R. (1993) Differential induction of distinct glutathioneS-transferases of Wheat by xenobiotics and by pathogen attack. Plant Physiol 102:1193–1201.
27.Nakano Y, Asada K. (1981) Hydrogen peroxide is scavenged by ascorbate-specific peroxidases in spinach chloroplasts. Plant Cell Physiol 22: 867–880.
28.Drazkiewicz M, Skorzy nska-Polit E, Krupa Z. (2003) Response of the ascorbate-glutathione cycle to excess copper in *Arabidopsis thaliana* (L.). Plant Sci 164: 95–202.
29.Badouin H, Gouzy J, Grassa CJ, Murat F, Staton SE, Cottret L, et al. (2017). The sunflower genome provides insights into oil metabolism, flowering and Asterid evolution. Nature 546(7656): 148–152.
30.Wu J, Zhao Q, Yang Q, Liu H, Li Q, Yi X, et al. (2016) Comparative transcriptomic analysis uncovers the complex genetic network for resistance to *Sclerotinia sclerotiorum* in *Brassica napus*. SCI Rep-UK 6:1-16.
31.Mwape VW, Mobegi FM, Regmi R, Newman TE, Kamphuis LG, Derbyshire MC. (2021) Analysis of differentially expressed *Sclerotinia sclerotiorum* genes during the interaction with moderately resistant and highly susceptible chickpea lines. BMC Genomics 22:1-14.
32.Chittem K, Yajima WR, Goswami RS, del Río Mendoza LE. (2020) Transcriptome analysis of the plant pathogen *Sclerotinia sclerotiorum* interaction with resistant and susceptible canola (*Brassica napus*) lines. PLoS ONE 15: e0229844.
33.Cosgrove DJ. (2005) Growth of the plant cell wall. Nat Rev Mol Cell Bio 6: 850-861.
34.Riou C, Hervé C, Pacquit V, Dabos P, Lescure B. (2002) Expression of an *Arabidopsis* lectin kinase receptor gene, lecRK-a1, is induced during senescence, wounding and in response to oligogalacturonic acids. Plant Physiol Bioch 40:431-438.
35.Zhao Y, Song D, Sun J, Li L. (2013) Populus endo-beta-mannanase PtrMAN6 plays a role in

coordinating cell wall remodeling with suppression of secondary wall thickening through generation of oligosaccharide signals. Plant J 74:473-485.

36. Wei W, Xiong Y, Zhu W, Wang N, Yang G, Peng F. (2016) Colletotrichum higginsianum mitogen-activated protein kinase ChMK1: role in growth, cell wall integrity, colony melanization, and pathogenicity. Front Microbiol 7:1212.

37. Madany MM, Saleh AM, Habeeb TH, Hozzei WN, AbdElgawad H. (2020) Silicon dioxide nanoparticles alleviate the threats of broomrape infection in tomato by inducing cell wall fortification and modulating ROS homeostasis. Environ Sci-NANO 7: 1415-1430.

38. Eynck C, Séguin-Swartz G, Clarke WE, Parkin IA. (2014) Monolignol biosynthesis is associated with resistance to *Sclerotinia sclerotiorum* in Camelina sativa. Mol Plant Pathol. 52;13: 887-899.

39. Cosgrove C. (2015) Comparing aquatic invertebrate communities on pin oak, cottonwood, and red maple leaf litter in vernal pools in northeastern Ohio. Kent State University.

40. Marowa P, Ding A, Kong Y. (2016) Expansins: roles in plant growth and potential applications in crop improvement. Plant Cell Rep 35;949-965.

41. Brasileiro AC, Lacorte C, Pereira BM, Oliveira TN, Ferreira DS, Mota AP, et al. (2021) Ectopic expression of an expansin-like B gene from wild Arachis enhances resistance to both abiotic and biotic stresses. Plant J 107:1681-1696.

42. Lombard V, Bernard T, Rancurel C, Brumer H, Coutinho PM, Henrissat B. (2010) A hierarchical classification of polysaccharide lyases for glycogenomics. Biochem J 432: 437-444.

43. Kubicek CP, Starr TL, Glass NL. (2014) Plant cell wall-degrading enzymes and their secretion in plant-pathogenic fungi. Annu Rev Phytopathol 52:427-451.

44. Yakoby N, Kobiler I, Dinoor A, Prusky D. (2000) pH regulation of pectate lyase secretion modulates the attack of Colletotrichum gloeosporioides on avocado fruits. Appl Environ Microb 66, 1026-1030.

45. Yang H, Yang Y L, Li G Q, Yu Q, Yang J. (2021) Identifications of immune-responsive genes for adaptative traits by comparative transcriptome analysis of spleen tissue from Kazakh and Suffolk sheep. Sci Rep-UK 11:1-16.

46. Wan J, He M, Hou Q, Zou L, Yang Y, Wei Y, Chen X. (2021) Cell wall associated immunity in plants. Stress 1:1-15.

47. Mittler R, Vanderauwera S, Suzuki N, Miller G A D, Tognetti V B, Vandepoele K, et al. (2011) ROS signaling: the new wave?. Trends Plant Sci 16: 300-309.

48. Apel K, Hirt H. (2004) Reactive oxygen species: metabolism, oxidative stress, and signaling transduction. Annu Rev Plant Biol 55:373.

49. Ding Y, Mei J, Chai Y, Yang W, Mao Y, Yan B, et al. (2020) *Sclerotinia sclerotiorum* utilizes host-derived copper for ROS detoxification and infection. Plos Pathog 16: e1008919.

50. Bowler C, Montagu M, Inze D. (1992) Superoxide dismutase and stress resistance. Annu Rev Plant Biol 43(1): 83-116.

51. Foyer CH, Noctor G. (2011) Ascorbate and glutathione: the heart of the redox hub. Plant Physiol 155: 2-18.

52. de Pinto MC, Tommasi F, De Gara L. (2002) Changes in the antioxidant systems as part of the signaling pathway responsible for the programmed cell death activated by nitric oxide and reactive oxygen species in tobacco Bright-Yellow 2 cells. Plant Physiol 130: 698-708.

53.Zhao J, Buchwaldt L, Rimmer SR, Sharpe A, McGregor L, Bekkaoui D, et al. (2009) Patterns of differential gene expression in Brassica napus cultivars infected with *Sclerotinia sclerotiorum*. Mol Plant Pathol 10:635-649.

54.Jain N, Menache I, Naor JS, Shepherd FB. (2012) Topology-aware vm migration in bandwidth oversubscribed datacenter networks. In International Colloquium on Automata, Languages, and Programming, Springer, Berlin, Heidelberg 586-597.

55.Noctor G, Foyer CH. (1998) Ascorbate and glutathione: keeping active oxygen under control. Annu Rev Plant Biol 49: 249-279.

56.Sakihama Y, Mano JI, Sano S, Asada K, Yamasaki H. (2000) Reduction of phenoxyl radicals mediated by monodehydroascorbate reductase. Biochem Bioph Res CO 279:949-954.

57.Kim I, Xu W, Reed JC. (2008) Cell death and endoplasmic reticulum stress: disease relevance and therapeutic opportunities. Nat Rev Drug Discov 7:1013-1030.

58.Zuo J, Li J. Molecular dissection of complex agronomic traits of rice: a team effort by Chinese scientists in recent years. Nalt Sci Rev. 2014;1:253-276.

59.Jones JDG, Dangl JL. (2006) The plant immune system. Nature 444(7117): 323-329.

60.Mengiste T. (2012) Plant immunity to necrotrophs. Annu Rev Phytopathol 50: 267-29.

61.Sun Y, Wang C, Yang B, Wu F, Hao X, Liang W, Niu F, Yan J, Zhang H, Wang B, Deyholos MK, Jiang YQ. (2014) Identification and functional analysis of mitogen-activated protein kinase kinase kinase (MAPKKK) genes in canola (*Brassica napus* L.). J Exp Bot 65:2171-2188.

62.Asai T, Tena G, Plotnikova J, Willmann MR, Chiu WL, Gomez-Gomez L, et al. (2002) MAP kinase signalling cascade in Arabidopsis innate immunity. Nature 415:977-983.

63.Wang Z, Ma LY, Cao J, Li YL, Ding LN, Zhu KM, et al. (2019) Recent advances in mechanisms of plant defense to *Sclerotinia sclerotiorum*. Front plant Sci 10: 1314.

64.Wang Z, Mao H, Dong C, Ji R, Cai L, Fu H et al. (2009) Overexpression of *Brassica napus* MPK4 enhances resistance to *Sclerotinia sclerotiorum* in oilseed rape. Mol Plant Microbe In 22:235-244.

65.Wang Z, Fang H, Chen Y, Chen K, Li G, Gu S, et al. (2014) Overexpression of BnWRKY33 in oilseed rape enhances resistance to *Sclerotinia sclerotiorum*. Mol Plant Pathol 15: 677-689.

66.Wang C, Yao J, Du X, Zhang Y, Sun Y, Rollins JA et al. (2015) The Arabidopsis mediator complex subunit16 is a key component of basal resistance against the necrotrophic fungal pathogen *Sclerotinia sclerotiorum*. Plant Physiol 169:856-872.

67.Takahashi F, Mizoguchi T, Yoshida R, Ichimura K, Shinozaki K. (2011) Calmodulin-dependent activation of MAP kinase for ROS homeostasis in *Arabidopsis*. Mol Cell 41(6):649–660.

68.Liu J, He B, Chen Q, Li J, Xiong Q, Yue G, et al. (2016) Direct synthesis of hydrogen peroxide from plasma-water interactions. Sci Rep-UK 6: 1-7.

69.McDowell JM, Dang JL. (2000) Signal transduction in the plant immune response. Trends Biochem Sci 25:79–82.

70.Martinez C, Blanc F, Le Claire E, Besnard O, Nicole M, Baccou JC. (2001) Salicylic acid and ethylene pathways are differentially activated in melon cotyledons by active or heat-denatured cellulase from Trichoderma longibrachiatum. Plant Physiol 127: 334–344.

71.Glazebrook J. (2005) Contrasting mechanisms of defense against biotrophic and necrotrophic pathogens. Annu Rev Phytopathol 43:205–227.

72.Broekaert IJ, Walker WA. (2006) Probiotics and chronic disease. J. Clin Gastroenterol

40:270-274.

73.Yang B. (2009) Characterization of the *Brassica napus*-fungal pathogen interaction. Doctoral dissertation, University of Alberta.

74. Asai T, Stone JM, Heard JE, Kovtun Y, Yorgey P, Sheen J, et al. (2000) Fumonisin B1–induced cell death in *Arabidopsis* protoplasts requires jasmonate-, ethylene-, and salicylate-dependent signaling pathways. Plant Cell 12:1823-1835.

Table 1 Activity changes in antioxidant enzymes in DS (B728) and DR (C6) sunflower inbred lines at 24 hpi by S. sclerotiorum infection.

Genotype	Treatment	SOD activity (ul·mg^{-1} protein·min^{-1})	POD activity (mmol·mg^{-1} protein·min^{-1})	CAT activity (mmol·mg^{-1} protein·min^{-1})	GST activity (μmol·mg^{-1} protein·min^{-1})	APX activity (mmol·mg^{-1} protein·min^{-1})	MDAR activity (mmol·mg^{-1} protein·min^{-1})
B728	Control	0.95±0.11b	14.35±1.35d	10.32±1.16d	20.64±2.02d	0.95±0.12a	5.37±0.92b
	inoculated	1.14±0.12b	24.36±2.02b	25.34±2.33b	31.27±2.33b	0.68±0.13b	3.28±0.84c
C6	Control	1.02±0.11b	18.37±1.39c	13.87±2.01c	25.64±2.12c	0.98±0.09a	6.11±0.81a
	inoculated	2.15±0.15a	30.11±2.87a	29.72±2.56a	35.69±2.24a	1.02±0.12a	6.59±0.72a

Table 2 Top 20 uniquely up-regulated genes in the DR genotype

Gene	Log_2 FC		Annotation
	B728	C6	
LOC110895548	-	9.24	Protein PLANT CADMIUM RESISTANCE 2
LOC110929546	-	8.93	Expansin-like B1
LOC110918750	-	8.25	Lignin-forming anionic peroxidase
LOC110885989	-	8.21	1-aminocyclopropane-1-carboxylate oxidase 3
LOC110886982	-	7.85	Berberine bridge enzyme-like 13
LOC110922271	-	7.83	EP1-like glycoprotein 4
LOC110879630	-	7.79	Probable L-gulonolactone oxidase 6
LOC110935776	-1.62	7.78	TMV resistance protein N
LOC110871630	-	7.73	Regulator of G-protein signaling 1
LOC110940063	-	7.70	Dirigent protein 21
LOC110928163	-	7.53	Cytochrome P450 87A3
LOC110877038	-	7.43	Cytokinin dehydrogenase 3
LOC110888580	-	7.39	Probable carboxylesterase 18
LOC110901085	-	7.38	-
LOC110934894	-	7.32	Ethylene-responsive transcription factor ERF098
LOC110925525	-	7.29	Hydroxycinnamoyltransferase 4
LOC110909848	-	7.25	Short-chain dehydrogenase reductase 2a
LOC110899788	-	7.21	1-aminocyclopropane-1-carboxylate oxidase homolog 1
LOC110945103	-	7.13	Cytochrome P450 71AV8
LOC110900121	-	7.06	Hydroquinone glucosyltransferase

Table 3 Top 20 uniquely down-regulated genes in the DR genotype

Gene	Log_2 FC		Annotation
	B728	C6	
LOC110885111	-5.70	-3.21	Proline-rich protein 4
LOC110930392	-5.55	-	Early light-induced protein 2
LOC110891330	-5.40	-	Soyasapogenol B glucuronide galactosyltransferase
LOC110869264	-4.92	-	Dehydration-responsive element-binding protein 1D
LOC110921230	-4.85	-2.19	-
LOC110909326	-4.54	-	-
LOC110869165	-4.54	-	Cyclin-U4-1
LOC110915744	-4.54	-	Leucine-rich repeat receptor protein kinase HPCA1
LOC110941770	-4.49	-	Bidirectional sugar transporter SWEET14
LOC110895020	-4.40	0.33	Probable galacturonosyltransferase-like 9
LOC110917579	-4.37	-0.67	Protein PHOSPHATE-INDUCED 1
LOC110908033	-4.19	-2.38	UPF0481 protein At3g47200
LOC110928088	-4.15	-	Protein SRC2
LOC110873198	-4.09	-1.73	Transcription repressor OFP1

LOC110929642	-4.06	-1.37	Receptor-like protein kinase THESEUS 1
LOC110912169	-4.06	-3.72	Probable inactive receptor kinase At1g48480
LOC110931795	-4.04	-2.49	-
LOC110864816	-4.01	-	GDSL esterase/lipase At4g01130
LOC110929231	-3.98	-1.20	Calmodulin-binding receptor kinase CaMRLK
LOC110868988	-3.94	-2.02	Uncharacterized protein At5g39865

Table 4 A list of transmembrane recognition receptors (PRRs) involved in plant-pathogen interaction pathway by KEGG analysis

Gene	Log_2 FC		Annotation
	B728	C6	
LOC110867023	5.60	6.46	Receptor-like protein 12
LOC110883941	4.02	3.60	Receptor-like protein 12
LOC110905009	2.29	1.71	Receptor-like protein EIX1
LOC110943528	2.50	1.42	Receptor-like protein EIX1
LOC110907898	5.24	3.84	Receptor-like protein EIX1
LOC110867197	1.66	1.20	Receptor-like protein EIX2
LOC110894990	6.67	4.51	Receptor-like protein EIX2
LOC110901887	3.27	2.31	Receptor-like protein EIX2
LOC110884455	5.47	3.79	Receptor-like protein EIX2 isoform X1
LOC110904904	3.57	1.12	Receptor-like protein EIX2 isoform X1
LOC110911286	-1.60	-1.26	Receptor-like protein 12
LOC110927258	7.29	-	Receptor-like protein 12
LOC110880537	5.50	-	Receptor-like protein 12
LOC110898370	2.33	-	Receptor-like protein 12
LOC110899583	6.89	-	Receptor-like protein 32
LOC110932987	7.90	-	Receptor-like protein 37
LOC110911343	2.13	-	Receptor-like protein EIX1
LOC110935701	2.81	-	Receptor-like protein EIX1 precursor
LOC110883962	2.01	-	Receptor-like protein EIX2
LOC110899544	3.10	-	Receptor-like protein EIX2
LOC110932925	7.28	-	Receptor-like protein EIX2
LOC110932954	6.64	-	Receptor-like protein EIX2
LOC110909891	1.04	-	Receptor-like protein EIX2
LOC110883961	2.45	-	Receptor-like protein EIX2
LOC110877393	1.16	-	Receptor-like protein EIX2 isoform X1
LOC110873395	-1.82	-	Receptor-like protein EIX2
LOC110880683	-	2.01	Receptor-like protein 12
LOC110904906	-	1.07	Receptor-like protein 12
LOC110872328	-	1.03	Receptor-like protein 12
LOC110867178	-	1.33	Receptor-like protein EIX1
LOC110872190	-	1.72	Receptor-like protein EIX2

LOC110932995	-	1.43	Receptor-like protein EIX2
LOC110927229	-	1.11	Receptor-like protein EIX2
LOC110924352	-	1.79	Receptor-like protein EIX2
LOC110887861	-	1.55	Receptor-like protein EIX2
LOC110880528	-	3.40	Receptor-like protein EIX2 isoform X1
LOC110867209	-	1.85	Receptor-like protein EIX2 isoform X1
LOC110867212	-	1.40	Receptor-like protein EIX2 isoform X1
LOC110872808	-	1.09	Receptor-like protein EIX2 isoform X1

Figure1

Click here to access/download;Figure;Fig1.tif

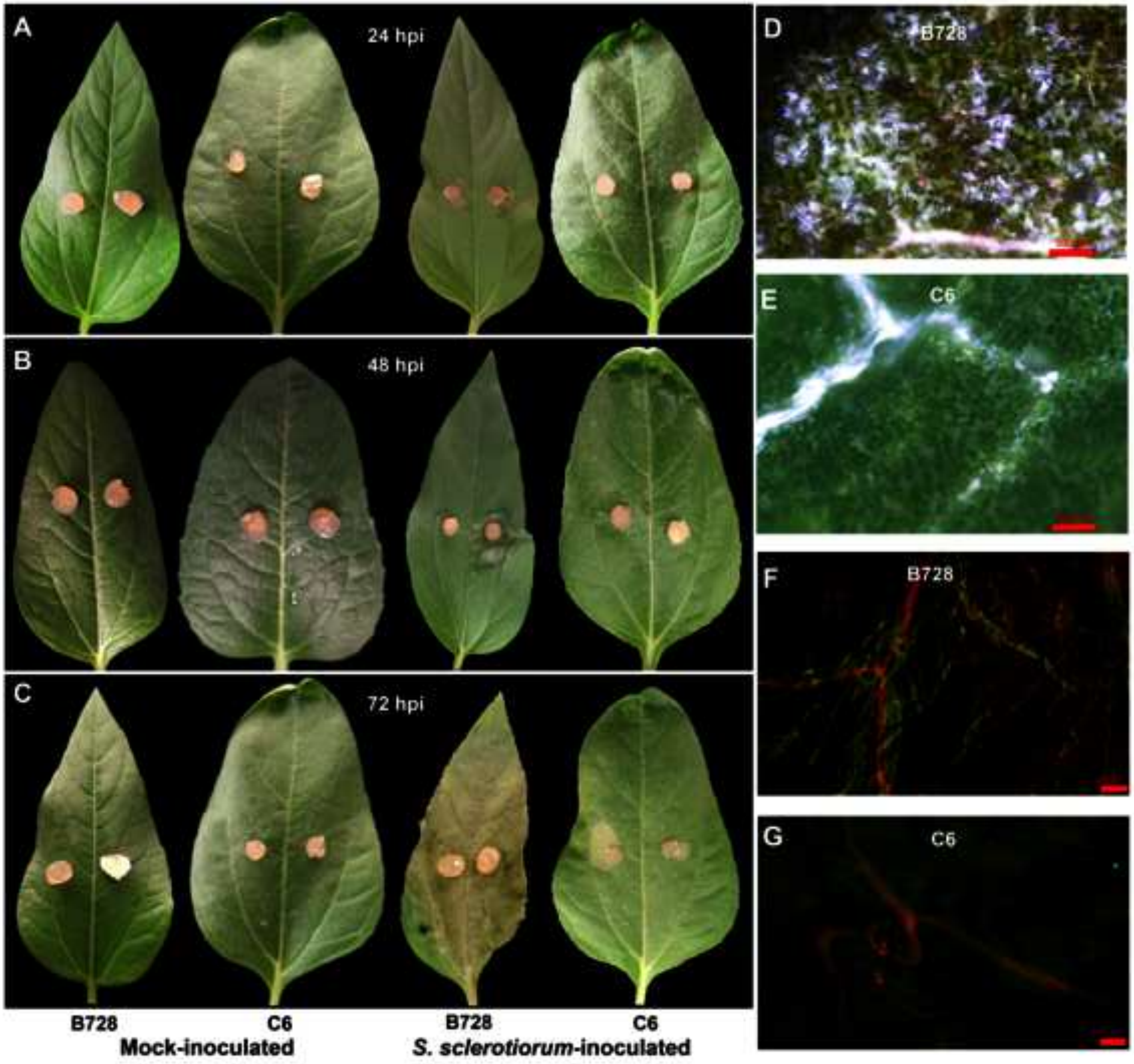

Figure2

Click here to access/download;Figure;Fig2.tif

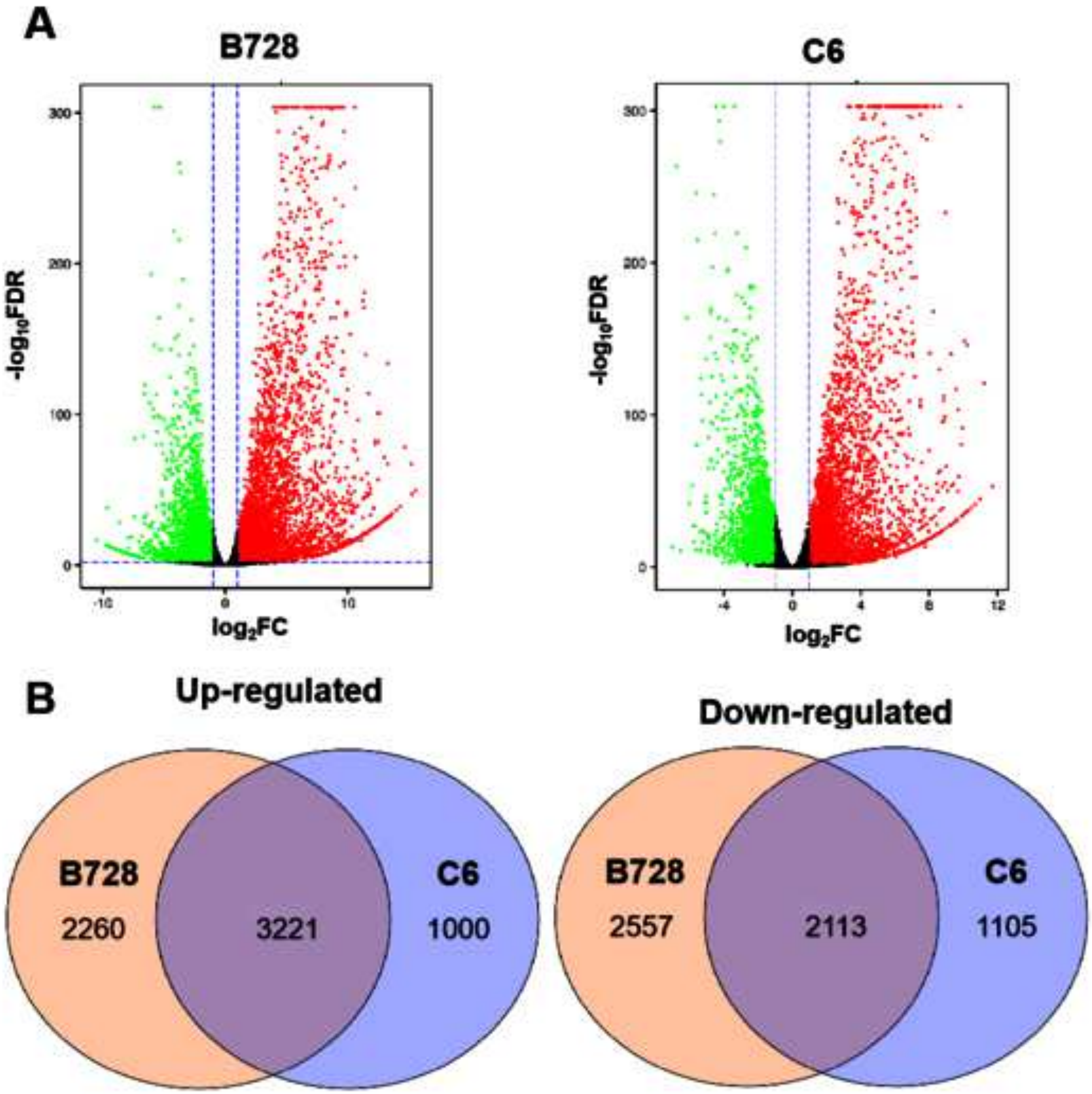

Figure3 Click here to access/download;Figure;Fig3.tif

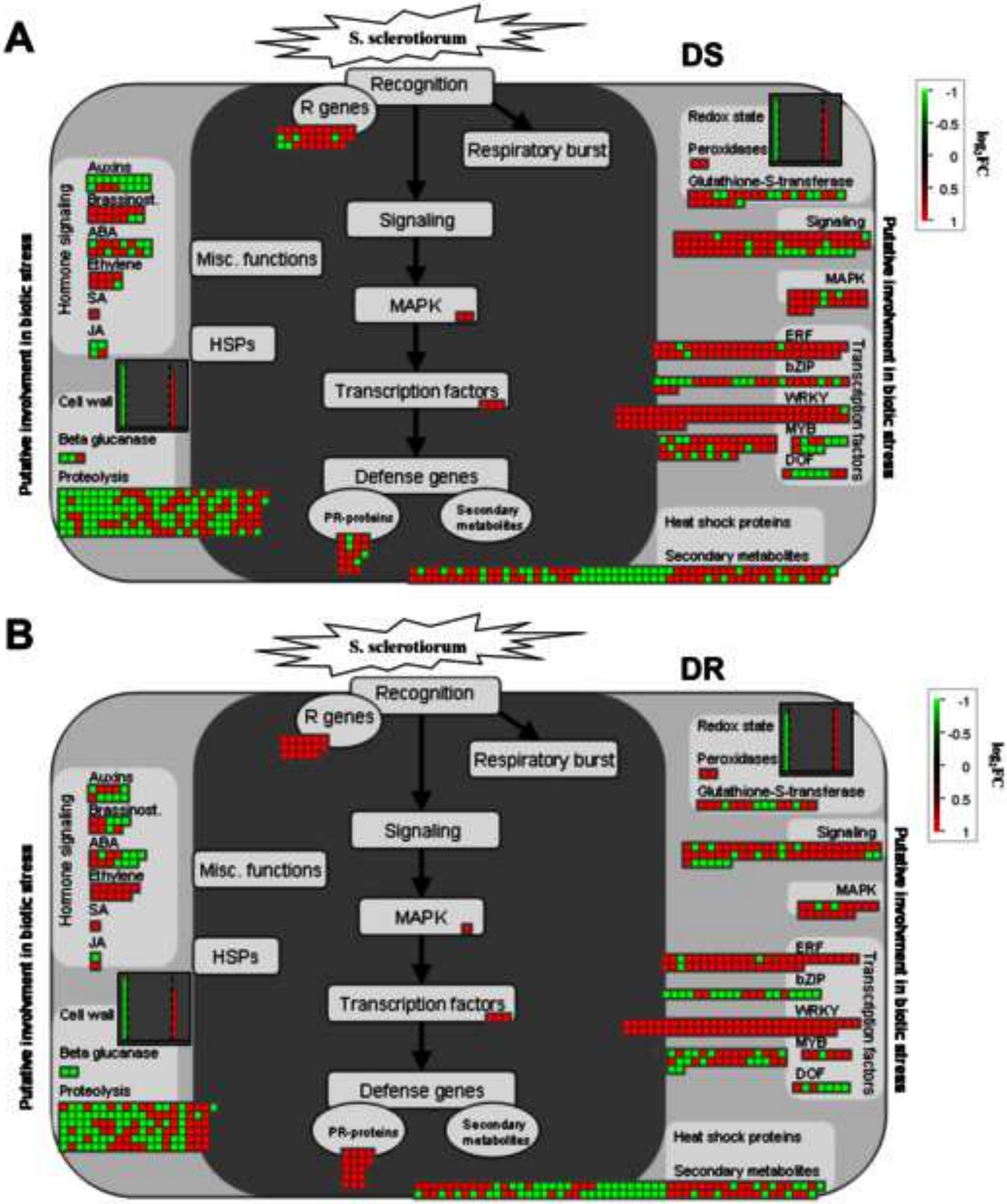

Figure4

Click here to access/download;Figure;Fig4.tif

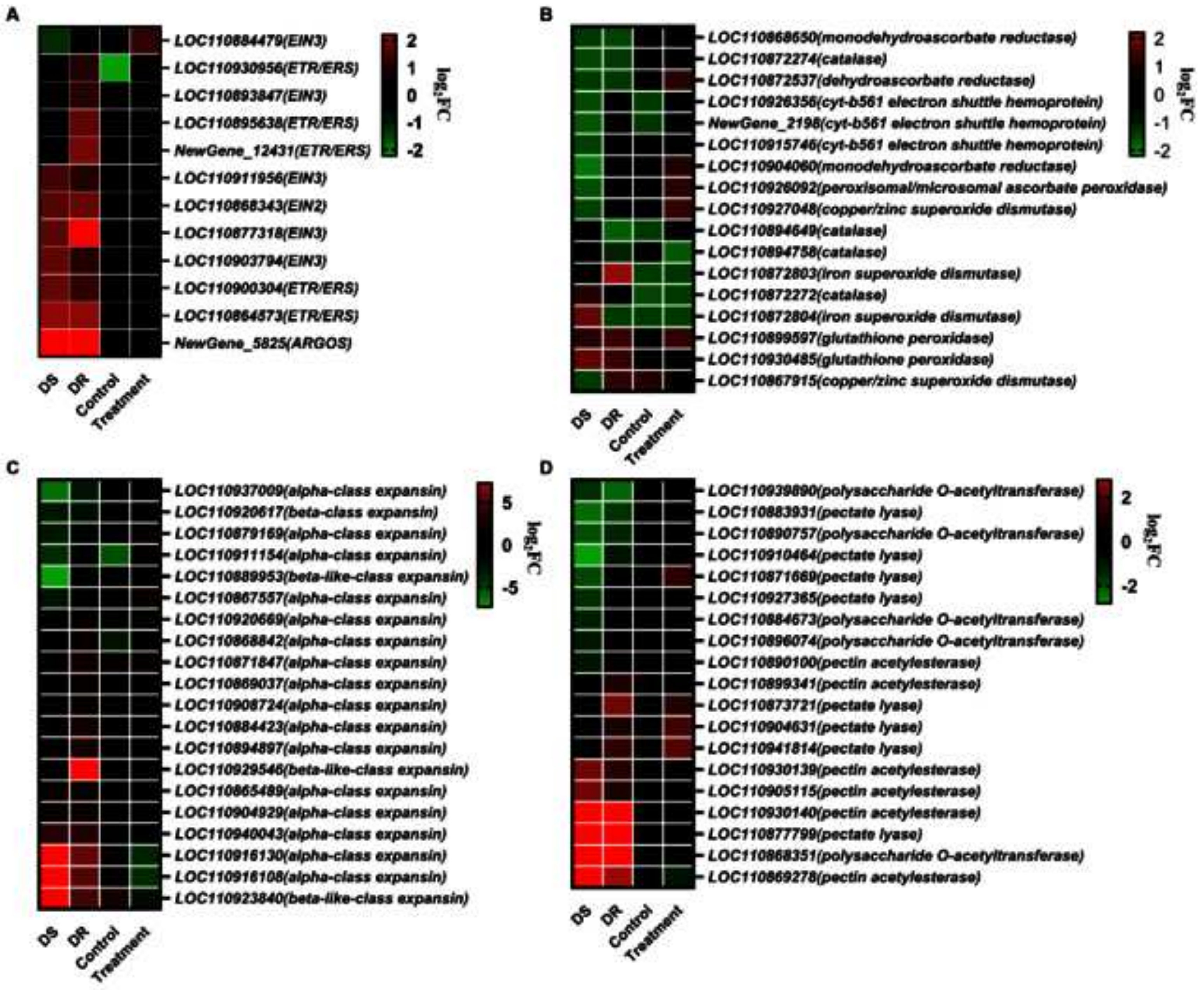

Figure5

Click here to access/download;Figure;Fig5.TIF

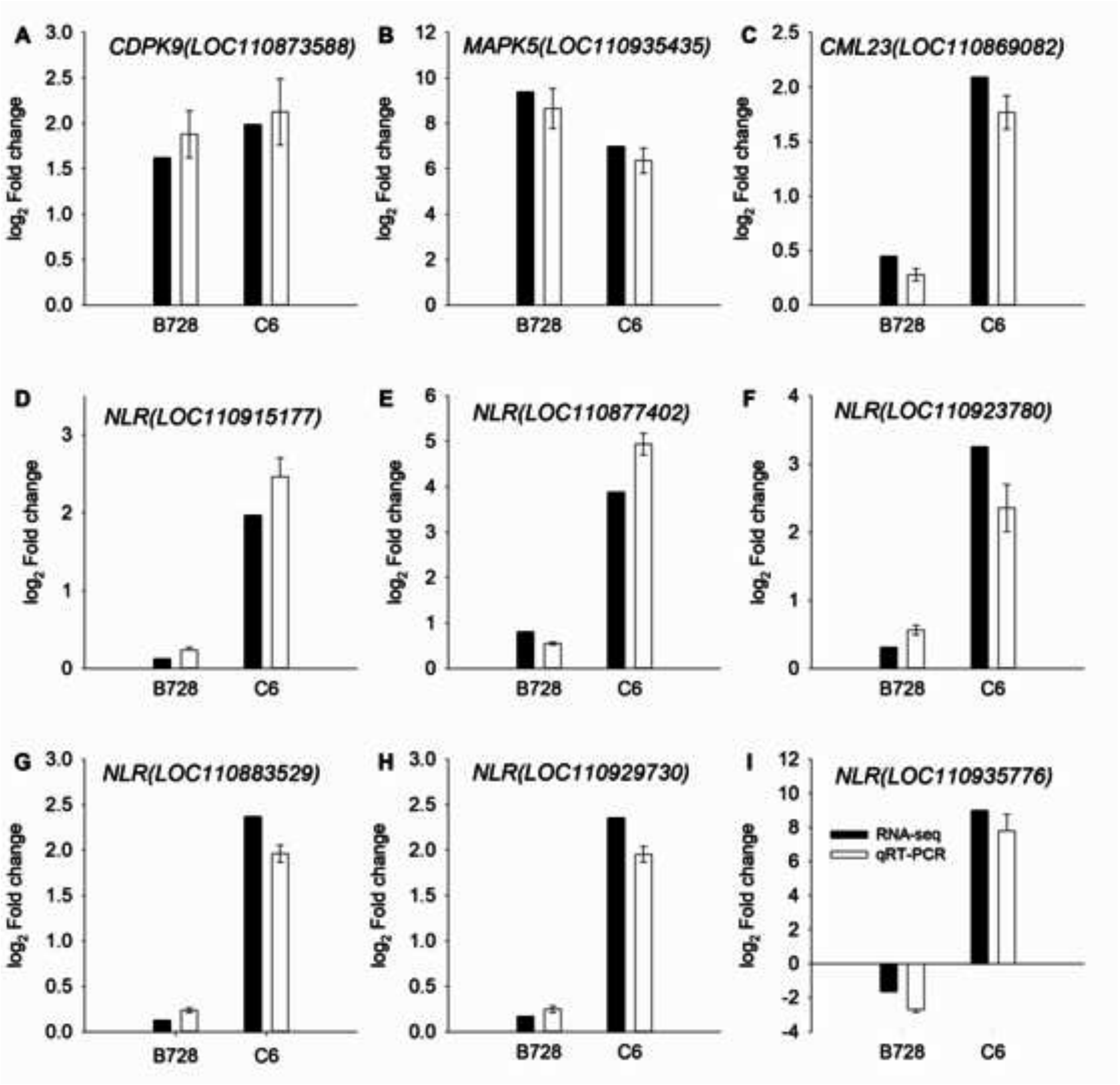

Figure6

Click here to access/download;Figure;Fig6.tif

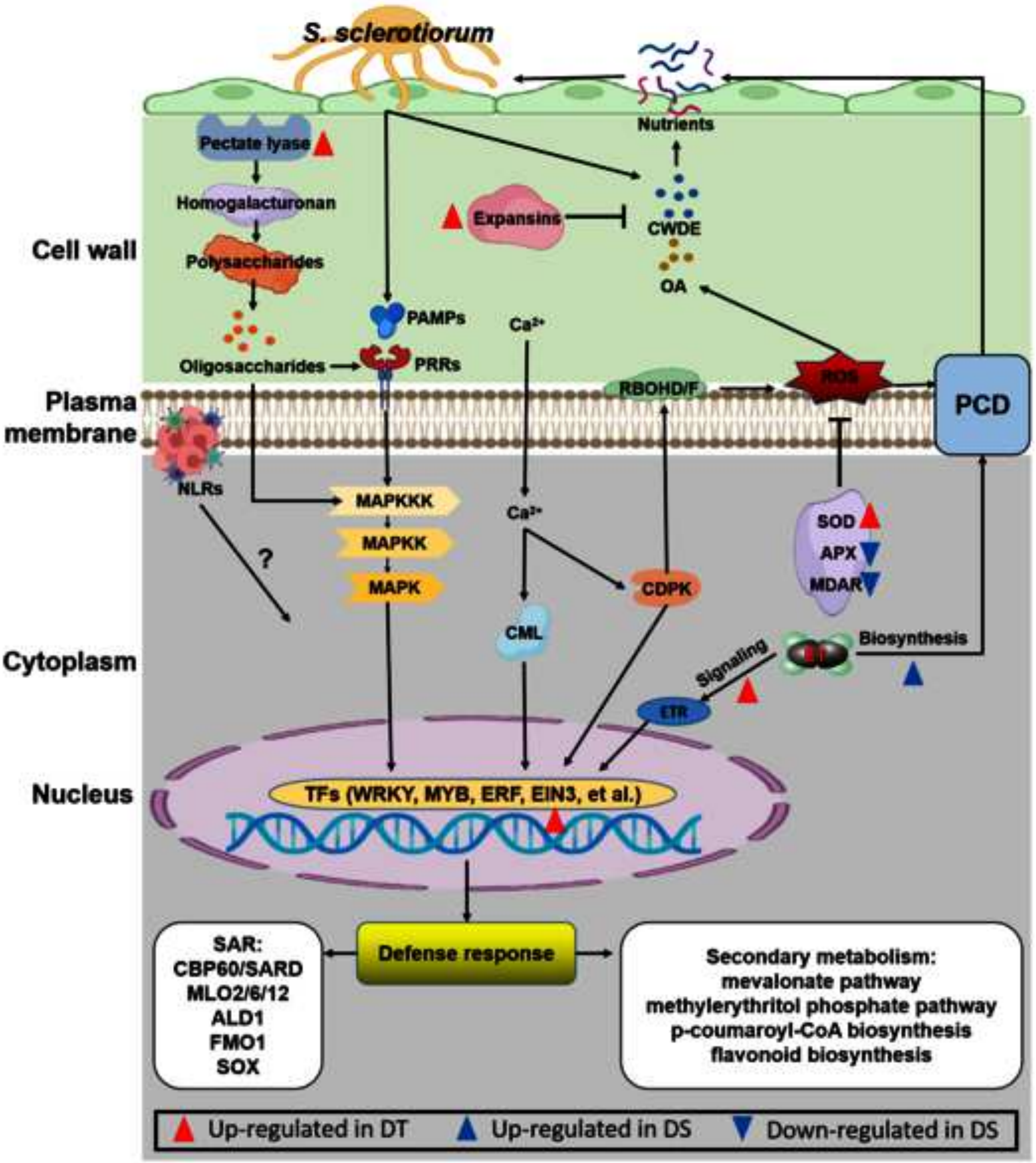

FigureS1

Click here to access/download;Figure;Fig S1.tif

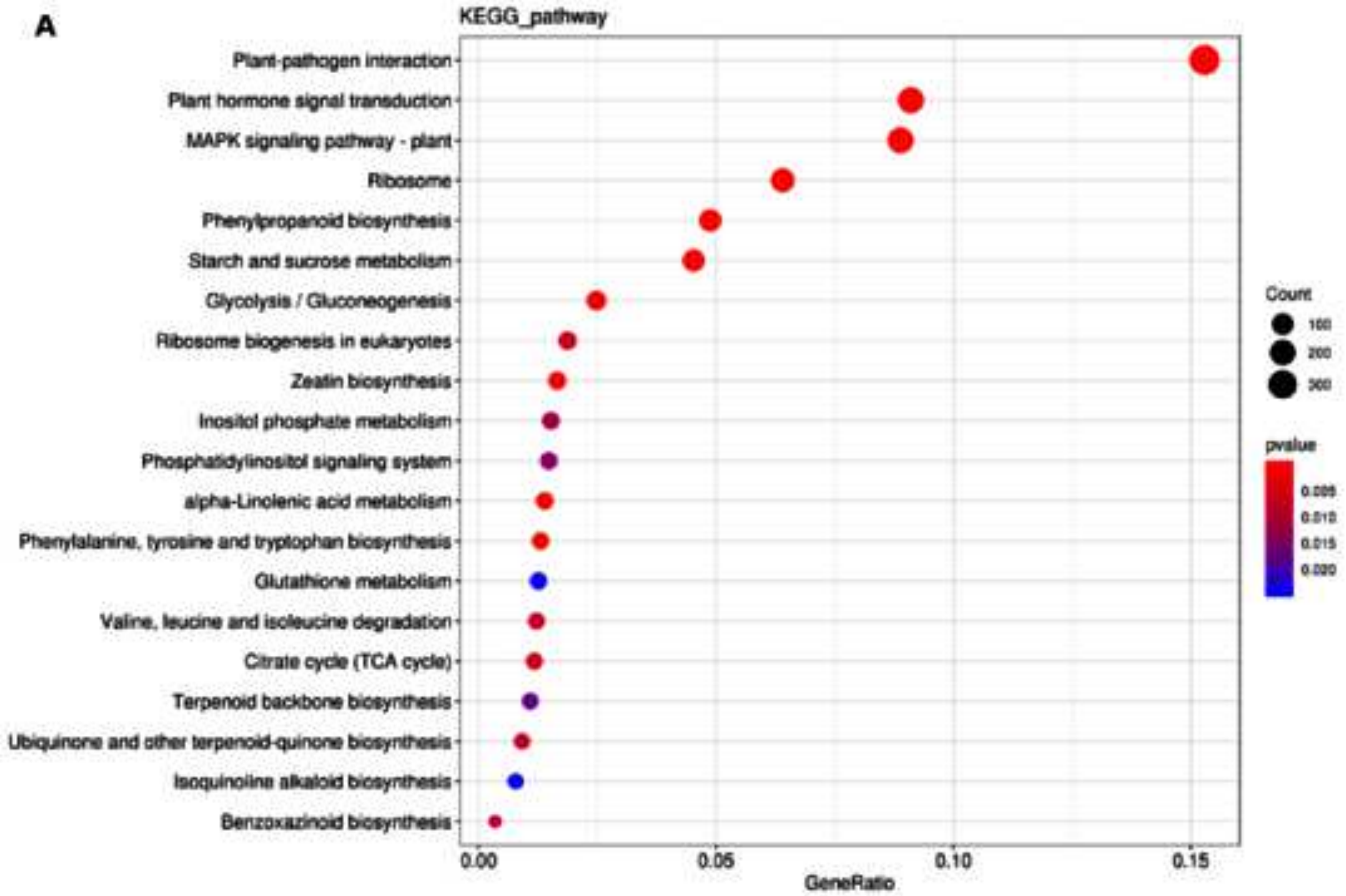

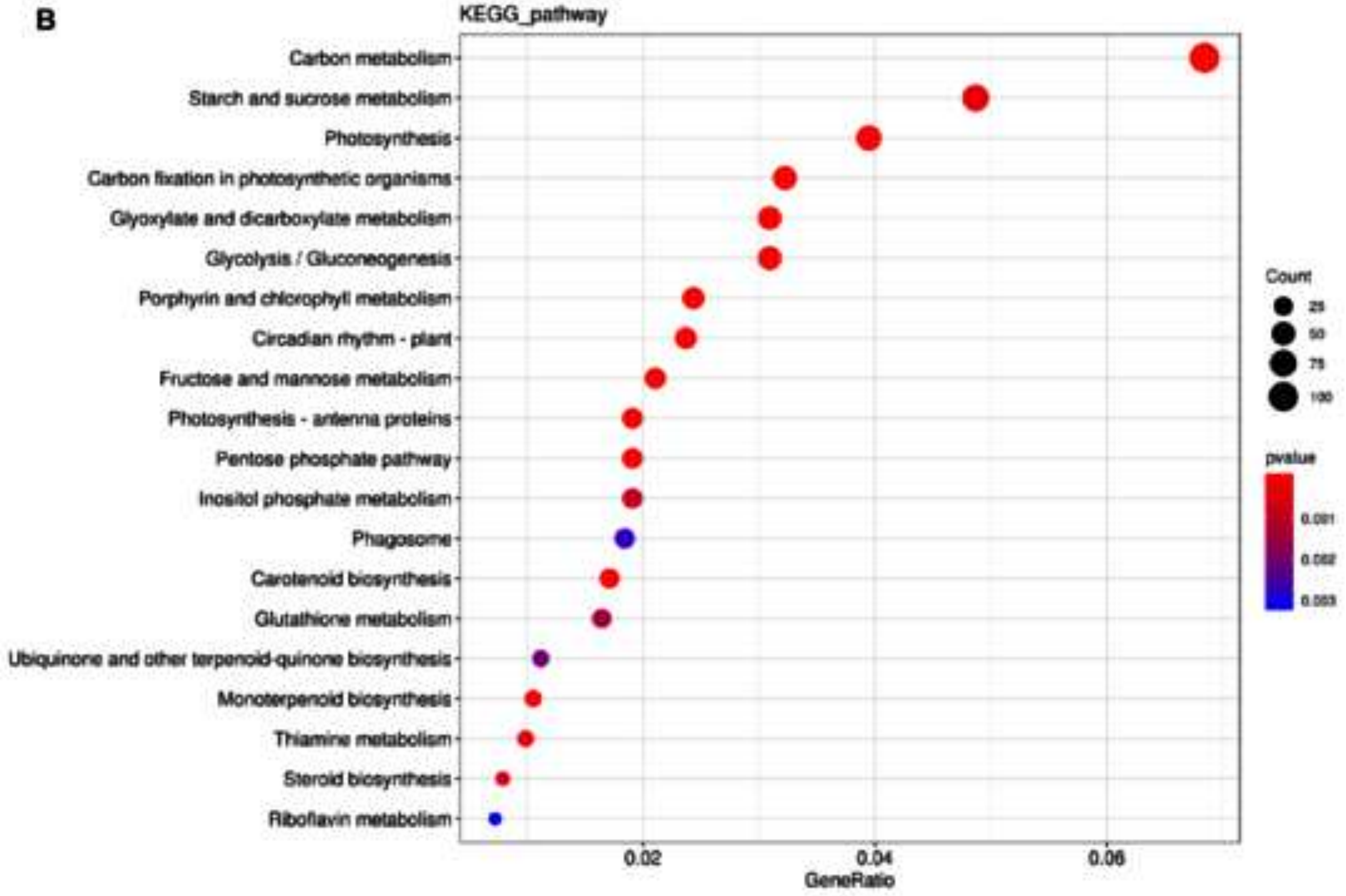

FigureS2

Click here to access/download;Figure;Fig S2.tif

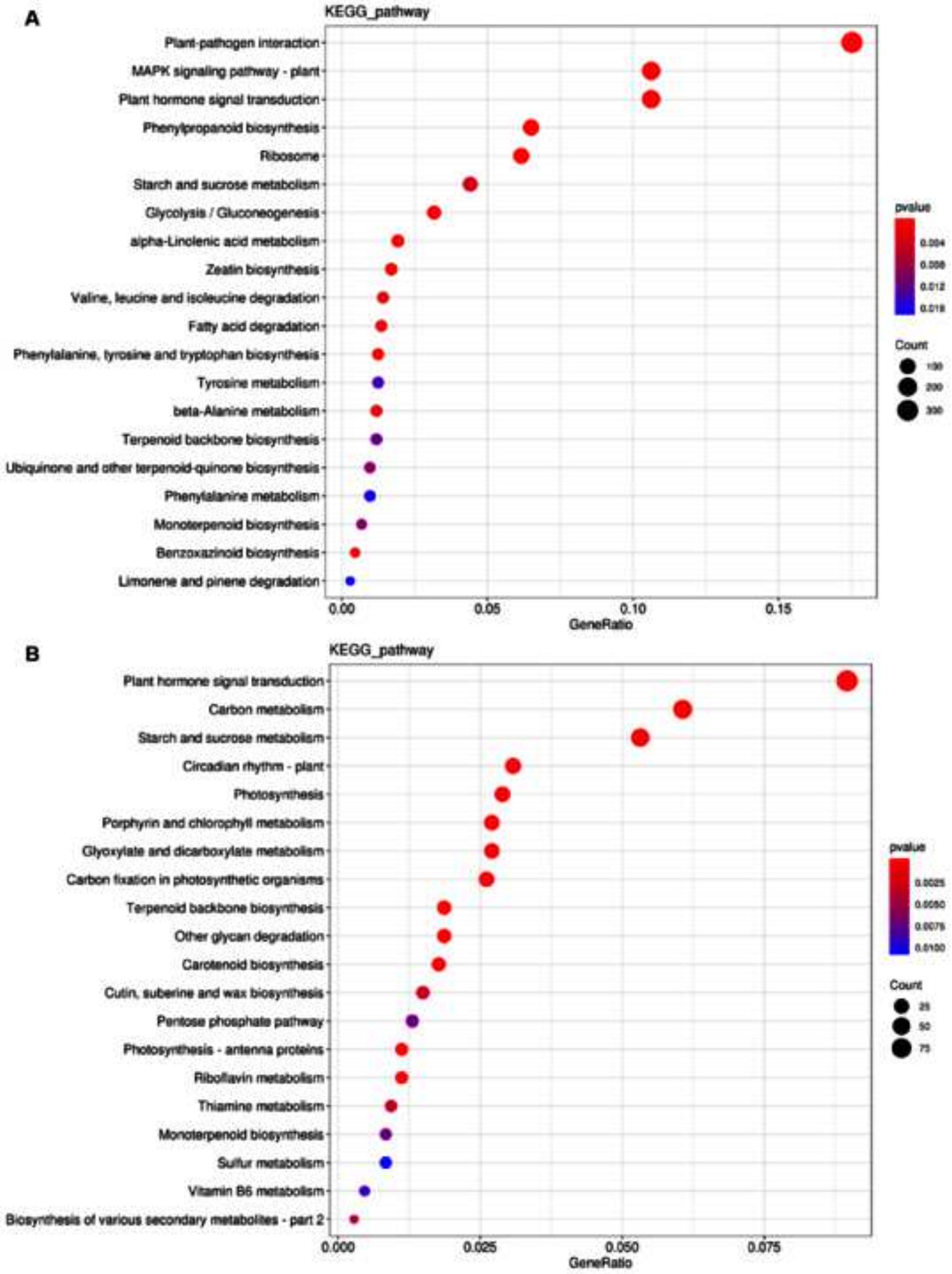

Click here to access/download
Supporting Information
Supporting Information-R2.xlsx

Comparative Transcriptome Analysis in Two Contrasting Genotypes for *Sclerotinia Sclerotiorum* Resistance in Sunflower

Mingzhu Zhao[1], Bing Yi[1], Xiaohong Liu[1], Dexing Wang[1], Dianxiu Song[1], Enyu Sun[1], Liangji Cui[1], Jingang Liu[1*], Liangshan Feng[2*]

,

[1]Institute of Crop Research, Liaoning Academy of Agricultural Sciences, Shenyang 110161, China

[2] Liaoning Academy of Agricultural Sciences, Shenyang 110000, China

* Corresponding author: Jingang Liu (jingangliu2022@163.com)

Liangshan Feng (fenglsh@163.com)

Abstract

Sclerotinia sclerotiorum as a necrotrophic fungus causes the devastating diseases in many important oilseed crops worldwide. The preferred strategy for controlling S. sclerotiorum is to develop resistant varieties, but the molecular mechanisms underlying *S. sclerotiorum* resistance remain poorly defined in sunflower (*Helianthus annuus*). Here, a comparative transcriptomic analysis was performed in leaves of two contrasting sunflower genotypes, disease susceptible (DS) B728 and disease resistant (DR) C6 after *S. sclerotiorum* inoculation. At 24 h post-inoculation, the DR genotype exhibited no visible growth of the hyphae as well as greater activity of superoxide dismutase activity (SOD), peroxidase (POD), catalase (CAT), glutathione-S-transferase (GST), ascorbate peroxidase (APX) and monodehydroascorbate reductase (MDAR) than DS genotype. A total of 10151 and 7439 differentially expressed genes (DEGs) were detected in DS and DR genotypes, respectively. Most of DEGs were enriched in cell wall organisation, protein kinase activity, hormone, transcription factor activities, redox homeostasis, immune response, and secondary metabolism. Differential expression of genes involved in expansins, pectate lyase activities, ethylene biosynthesis and signaling and antioxidant activity after *S. sclerotiorum* infection could potentially be responsible for the differential resistance among two genotypes. In summary, these finding provide additional insights into the potential molecular mechanisms of *S. sclerotiorum*'s defense response and facilitate the breeding of *Sclerotinia*-resistant sunflower varieties.

Keywords: Differentially expressed genes; Molecular mechanisms; Sunflower (*Helianthus annuus*); *Sclerotinia sclerotiorum*; Transcriptomic

Introduction

Sunfower (*Helianthus annuus*), is the third oilseed crop in the world, primarily for production of high-quality cooking oil and confectionary products and bird food, among others [1]. The occurrence of *Sclerotinia* and the lack of Sclerotinia-resistant varieties led to the decline of the planting area and yield in sunflower. *Sclerotinia sclerotiorum* (Lib.) de Bary is a kind of necrotrophic fungus, which infects over 400 species of plants worldwide including important oilseed crops, such as soybean, canola and sunflower [2-4]. Crop productivity and oil quality can be negatively affected by stem rot, root rot and head rot of plant at different developmental stages [5-6]. Fungal pathogens penetrates the host cuticle first forms an infection pad through mechanical pressure or secreting keratinase, then produce oxalic acid, toxic compounds, cell-wall-degrading enzymes and develop water-soaked lesions, tissue necrosis and finally bear sclerotia [7]. Although a few fungicides are available to manage this disease, the little efficiencies, the environmental costs cannot be ignored [8]. Breeding and cultivating resistant varieties are the best strategy to control *S. sclerotiorum* [9-10]. For the effective breeding of *Sclerotinia*-resistant varieties, therefore, it is essential to understand the genetic and molecular basis of the interactions of *S. sclerotiorum* with its host crops.

删除: (OA)

删除: (toxins)

删除: (CWDE)

Pathogen-associated molecular pattern-triggered immunity (PTI) and effector-triggered immunity (ETI) as the major immune systems in plants have been evolved to cope with pathogens [11]. For pathogen recognition, PTI uses cell-surface pattern recognition receptors (PRRs) that respond to pathogen-associated molecular pattern. The early reactions of PTI include calcium influx, production of reactive oxygen species (ROS), and activation of mitogen activated protein kinases (MAPKs) to trigger transcriptional processes regulated by plant transcription factors [12]. PTI plays an important role in plant resistance to *S. sclerotiorum* [13]. However, adapted pathogens able to suppress PTI may be recognized by plant intracellular resistance proteins of the nucleotide-binding and leucine-rich repeat (NLR) family, encoded by resistance (R) genes, leading to ETI [14, 15]. ETI triggers a series of defense responses in the host plant, most commonly at the infection site causing programmed cell death and anaphylaxis (thereby limiting the growth, reproduction, and expansion of the pathogen), and this is a very effective way for biotrophs fungi, but for necrotrophs fungi, it can kill the host cells and feed on the contents, while biotrophs

删除: (PAMP)

删除: PAMP

删除: (PCD)

complete their life cycle depending on the living host cells [16]. To date, no PRRs or R proteins involved in recognizing *S. sclerotiorum* in host plants have been observed [17, 18].

Transcriptional profling at the site of infection provides an efficient means to reflect the natural epidemiologic cycle among responses between resistant and susceptible varieties [19]. Previous studies characterized the transcriptomic changes in soybean, *Brassica napus*, and *Arabidopsis thaliana* during the defense responses to *S. sclerotiorum*, which involves rapid induction of key pathogen responsive genes, including glucanases, chitinases, peroxidises and WRKY transcription factor family, pathogenesis related proteins, as well as genes related to signal transduction, cellular redox state, cell wall composition and hormone signaling pathways [20, 21]. However, the molecular regulatory mechanisms of sunflower in response to *S. sclerotiorum* remain poorly understood. In this study, we performed a leaf transcriptomic analysis of resistant and susceptible sunflower to determine the defense responses to *S. sclerotiorum* using RNA sequencing (RNA-seq). This work could provide new insight into understanding of the molecular mechanisms underlying the interaction between *S. sclerotiorum* and sunflower.

删除: (PR)

Materials and methods

Plant materials, inoculation and sampling

Both DR genotype C6 and DS genotype B728 are oilseed sunflower inbred lines which were bred by Liaoning Academy of Agricultural Sciences. The plants of these lines, which had similar growth periods, were cultivated in a growth chamber under a 16 h/8 h light/dark cycle (light intensity 4000 Lux) at 25℃. The infected inflorescences were sampled from field-grown sunflowers in Shenyang, Liaoning Province, China, to isolate and purify the *S. sclerotiorum*. The strain of *S. sclerotiorum* in present study was isolated and maintained on potato dextrose agar medium. A single mycelial plug (3 mm diameter) was cut from the culture to place at the center of a new potato dextrose agar plate for incubation at 25℃ for 5 d in the dark. A total of ten mycelial agar plugs were collected from the edge of the colony for incubation in another new potato dextrose agar plate with the same conditions. Mycelial agar plugs were excised from the margin of a growing colony and used to inoculate sunflower leaves of 6 week old, while potato dextrose agar

删除: (PDA)

删除: PDA

删除: ℃

删除: PDA

删除: PDA

agar plugs without fungus was used as a mock-inoculated control. A randomized complete-block was used in the experimental design with three replications, and each replicate contained 20 plants. As the visible lesions appeared in DS genotype rather than DR genotype at 24 h post-inoculation (hpi), then the 20 leaves samples within the range of 0.5 cm bordering the extending lesion from inoculated and mock-inoculated plants were harvested for each replicate. Immediately after sampling, all samples were washed with sterilized water and placed into storage tubes, frozen with liquid nitrogen, and then stored at -80℃ for biochemical assays and RNA isolation.

删除: A

删除: ℃

Microscopic observation

To observe the growth of *S. sclerotiorum* in sunflower, the leaves samples from inoculated plants of both genotypes were sampled at 24 hpi for wheat germ agglutinin (WGA) staining [22]. Single thin shavings were cut from the leaves samples, and placed in 2 ml microcentrifuge tubes. Then 10% KOH was used to soake the samples at room temperature, which had been rinsed once with distilled water. After rinsing three times with distilled water later, the samples were incubated with WGA stain solution, which was consisting of 10 μg/ml WGA, 0.02% (v/v) Tween 20, 10 mM phosphate-buffered saline, pH 7.4. By using vacuum infiltration for 30 min, the samples were washing three times with phosphate-buffered saline. Then, we finally observed the samples under a FV 1000 fluorescence microscope (OLYMPUS) with excitation at 488 nm and emission at 500 to 540 nm.

Antioxidant enzyme extraction and activity assays

To obtain the enzymatic extract, frozen leaves (500 mg) from each sample was ground in liquid nitrogen to a fine powder. The tissue were grinded with borate buffer, and then the homogenate volume reached a final volume of 10 mL with borate buffer and centrifuged at 10,000 rpm for 15 min at 4℃.

The superoxide dismutase activity (SOD) activity was determined as of the ability of photochemical reduction of nitrotetrazolium according to the method described by Giannopolitis and Ries [23]. The peroxidase (POD) activity was carried out by the method of Shannon et al [24]. The activity of catalase (CAT) was determined according to the method of Aebi [25]. The

删除: ℃

删除: superoxide dismutase activity (SOD), peroxidase (POD), catalase (CAT), glutathione-S-transferase (GST), ascorbate peroxidase (APX) and monodehydroascorbate reductase (MDAR)

删除: SOD

删除: POD

删除: CAT

glutathione-S-transferase (GST) activity was measured as described by Mauch and Dudler [26]. The ascorbate peroxidase (APX) activity was obtained according to Nakano and Asada [27], using ascorbic acid as a substrate. The monodehydroascorbate reductase (MDAR) activity was assayed according to Drazkiewicz et al [28]. All the enzymatic analyses were performed in triplicate.

删除: GST

删除: APX

删除: MDAR

RNA sequencing and analysis of RNA-seq data

Sunflower leaves were extracted using the RNAprep Pure Plant Kit (TIANGEN Biotech, Beijing, China) according to the instructions provided by the manufacturer. RNA samples were used to measure the concentration and purity in NanoDrop 2000 (Thermo Fisher Scientific, Wilmington, DE). The integrity of RNA samples was measured by using the RNA Nano 6000 Assay Kit of the Agilent Bioanalyzer 2100 system (Agilent Technologies, CA, USA). In this study, we used RNA samples with a 260/280 ratio of 1.96-2.17, a 28S/18S ratio of 1.0, and a RNA integrity number greater than 8.0 to prepare RNA-Seq libraries using NEBNext UltraTM RNA Library Prep Kit (NEB, USA) according to the manufacturer’s instructions. The clustering of the index-coded samples was performed on a cBot Cluster Generation System using TruSeq PE Cluster Kit v4-cBot-HS (Illumia). Finally, the RNA sequencing was performed on IlluminahpiiSeq 2500 platform by the Biomarker Technology, Qingdao, China.

Sequencing data were analyzed with the help of a bioinformatic pipeline tool, the BMKCloud platform (www.biocloud.net). Raw reads of fastq format were firstly processed through in-house perl scripts. In this step, the clean reads were obtained by removing adapter-containing reads, ploy-N-containing reads, and low quality reads from raw data. The Q20, Q30, GC-content and sequence duplication level of the clean data were calculated. All the downstream analyses were based on clean data with high quality. The adaptor sequences and low-quality sequence reads were removed from the data sets. Raw sequences were transformed into clean reads after data processing. These clean reads were then mapped to the reference genome sequence. Only reads with a perfect match or one mismatch were further analyzed and annotated based on the sunflower reference genome. The HanXRQr1.0 genome was used as reference in the present study [29]. Then, Hisat2 tools soft were used to map with reference genome. The StringTie Reference Annotation Based Transcript (RABT) assembly method was used to construct and identify both

删除: For all downstream analyses,

删除: as

删除: HISAT2 and StringTie aligned and assembled the filteted reads to *Helianthus annuus* reference genome.

known and novel transcripts from Hisat2 alignment results. The gene expression levels are reflected by fragments per kilobase of transcript per million fragments mapped (FPKM). Genes with low expression (FPKM <1) will be eliminated by default in the analysis process. An analysis of differential expression was carried out using the DESeq2 package. The false discovery rate (FDR) was controlled by using the Benjamini and Hochberg's approach to adjust the resulting P values. The genes were identified as DEGs when the FDR ≦ 0.05 as well as the fold change ≧ |2|.

Functional classification and pathway analysis

Enrichment of Gene Ontology (GO) terms of the DEGs was implemented by AgriGO v2.0 (http://systemsbiology.cau.edu.cn/agri-GOv2/) with hypergeometric statistical test and Hocberg (FDR). The Kyoto Encyclopedia of Genes and Genome (KEGG, http://www.genome.jp/kegg/) was utilized for pathway enrichment analysis of DEGs. The overview of metabolic pathways in which DEGs involved in response to *S. sclerotiorum* were visualized by using MapMan software. In the MapMan software, the natural log of the ratio of the experimental mean to the control mean of the above called DEGs was taken in input files. Final analyses were performed with MapMan version 3.6.0 including the automatic application of the Wilcoxon rank sum test. Mapping files were exported from MapMan.

删除: analyses

Validation of RNA-seq analysis by qPCR

The extracted RNA was also used for qRT-PCR analysis to confirm the DEG results from RNA-seq. The same samples for RNA-seq were also used to extract the total RNA, which was further used to synthesize the cDNA. The qRT-PCR was conducted using ChamQ Universal SYBR qPCR Master Mix (Vazyme Biotech co., ltd, NanJing, China) on a Light Cycler 480 II (Roche, Basel, Switzerland). Primer sequences are described in S1 Table. The cycle threshold (CT) values obtained from three replicates and four technical replicates. The fold changes were calculated by $2^{-\Delta\Delta CT}$. $\Delta\Delta CT= (CT_{target}-CT_{action})_{treatment} - (CT_{target}-CT_{action})_{control}$. Additionally, the results of qRT-PCR were presented as log_2 fold changes to assess the accuracy of the RNA-seq.

删除:

Results

Symptoms and physiological traits in leaves of sunflower inoculated with *S. sclerotiorum*

Leaves from 6-week-old plants of two sunflower inbred lines, DR genotype C6 and DS genotype B728, were inoculated with isolated *S. sclerotiorum* using mycelial agar plugs. Visible lesions appeared in DS genotype at 24 hpi (Fig. 1A) and in DR genotype at approximately 72 hpi (Fig. 1C). The lesions developed more rapidly in DS genotype than in DR genotype. After 48 hpi (Fig. 1B&C), the lesions on the leaves of DR genotype were significantly smaller than those on those of DS genotype. By electron microscopy, we found chlorosis phenomenon in the leaf tissue of DS genotype (Fig. 1D) rather than DR genotype at 24 hpi (Fig. 1E). Microscopic assessments of the hyphae further showed that the pathogen was well established in the host tissue and led to extensive mycelial growth in DS genotype at 24 hpi (Fig. 1F), while no visible growth of the hyphae was found in DR genotype (Fig. 1G).

The physiological response of leaves to *S. sclerotiorum* inoculation was further evaluated based on the activity of antioxidant enzymes, such as SOD, POD, CAT, GST, APX and MDAR. These data are shown in Table 1. Following infection, the activity of POD, CAT and GST were increased significantly in both genotype, while the SOD activity was increased significantly in DR genotype rather than DS genotype. APX and MDAR activity in DS genotype was decreased significantly by *S. sclerotiorum* infection, while DR genotype showed non-significant change. *S. sclerotiorum* infection, caused higher activity of the enzymes relative to the DS genotype.

RNA-seq analysis and identification of DEGs

By RNA-seq, average of 43 million raw reads were obtained for each sample, and 74.8-91.8% of trimmed reads were mapped to the reference genome. With a cutoff value of 1, a total of 22027 and 22579 genes were expressed in mock-inoculated samples of DS and DR genotypes, respectively, while 20711 and 23636 in the *S. sclerotiorum*-inoculated samples. The DR genotype constitutively expressed more genes than did the DS genotype.

删除: superoxide dismutase activity (
删除:)
删除: peroxidase (
删除:)
删除: catalase (
删除:)
删除: glutathione-S-transferase (
删除:)
删除: ascorbate peroxidase (
删除:)
删除: monodehydroascorbate reductase (
删除:)
删除: A
删除: 36969
删除: 37854
删除: 36363
删除: 39538

In order to determine whether DS and DR genotypes respond differently to *S. sclerotiorum* inoculation, DEGs were obtained by comparing gene expression levels between *S. sclerotiorum*- and mock-inoculated samples of the same genotype. In DS genotype, 10151 DEGs were identified between *S. sclerotiorum*- and mock-inoculated samples: 5481 up-regulated and 4670 down-regulated, whereas the same analysis in the DR genotype revealed only 7439 DEGs: 4221 up-regulated and 3218 down-regulated (Fig. 2A). Venn diagrams showed that 2260 and 1000 genes were uniquely up-regulated in DS and DR genotypes, respectively, while 2557 and 1105 were uniquely down-regulated, respectively (Fig. 2B). In addition, 119 genes were only induced in DS genotype after *S. sclerotiorum* infection but have constitutively expressed in the DR genotype (S2 Table).

删除:

Gene ontology (GO) enrichment analysis of DEGs

删除: By using gene ontology (GO) classification (Fig. S1), a total of 8331 (4690 up-regulated and 3461 down-regulated) and 6148 (3499 up-regulated, and 2649 down-regulated) DEGs in DS and DR genotypes, respectively, were assigned GO numbers and classified into the three principal categories of biological process (BP), cellular component (CC), and molecular function (MF). In both DS and DR genotypes, the “cellular process”, “metabolic process”, “single-organism process”, and “biological regulation” were the most frequent in the BP category; In the CC category, “cell”, “cell part”, “cell organelle’, and “membrane” were most frequent; In the MF category, the most frequently represented were “binding” and “catalytic activity”, followed by “transporter activity” and “nucleic acid bingding transcription factor activity”.

AgriGO software was used to calculate the singular enrichment analysis (SEA) for the up- and down-regulated genes in DS genotypes, highlighted 126 and 134 GO significantly enriched terms, respectively (S3-4 Table), while 98 and 88 GO terms were significantly enriched the up- and down-regulated genes in DR genotype (S5-6 Table). We identified 249 and 282 common GO terms that were enriched in both the genotypes from up- and down-regulated genes in response to *S. sclerotiorum*, respectively (S7-8 Table), which play an important role in the disease resistance of sunflower. For the up-regulated genes, 155 GO terms including protein kinase activity, protein autophosphorylation, response to oxidative stress, chitin catabolic process, immune response, cell surface receptor signaling pathway, nucleotide binding, plasma membrane and peroxidase activity were more enriched in DR genotype than in DS genotype (S7 Table). Among the 145 GO terms for the down-regulated genes, the more enriched terms in DS genotype than in DR genotype included photorespiration, chlorophyll biosynthetic process, photosynthesis, chloroplast, plastid, thylakoid, organelle subcompartment, oxidoreductase activity and chlorophyll binding (S8 Table).

The SEA analysis was carried out on the uniquely up- and down-regulated genes in DS genotypes. Among the uniquely up-regulated genes 26 GO terms were significantly enriched: “intracellular protein transport” in the BP category, “clathrin vesicle coat” in CC category, and “GTPase activity” in MF category (S9 Table). “Carbohydrate metabolic process” in BP category,

删除: the

删除: Fig. 3

删除: top twenty

删除: and

删除: Fig. 3A

删除: twenty

删除: and

删除: Fig. 3B

删除:

"vacuole" in CC category and "metal ion binding" in MF category were the most enriched GO terms among the 118 identified from unique down-regulated genes in the DS genotype (S10 Table).

The SEA analysis was also carried out on the uniquely up- and down-regulated genes in DR genotypes. Among the uniquely up-regulated genes 63 GO terms were significantly enriched: "cellular polysaccharide metabolic process" in the BP category, "cellular polysaccharide biosynthetic process" in CC category, and "cell morphogenesis" in MF category (S11 Table). "Cellular component organization" in BP category, "plasma membrane" in CC category and "transcription factor activity, sequence-specific DNA binding" in MF category were the most enriched GO terms among the 16 identified from unique down-regulated genes in the DR genotype (S12 Table).

删除: 7

删除: 8

Top twenty uniquely up- (log_2FC >7) or down-regulated (log_2FC <-4) genes were identified in the DR genotypes, respectively (Table 2 and 3), most of which were enriched in the above uniquely up- or down-regulated GO terms. These uniquely up-regulated genes in the DR genotype were annotated as cytokinin dehydrogenase, cytochrome P450, expansin, transcription factor ERF, TMV resistance protein, etc (Table 2). These uniquely down-regulated genes in DS genotype were annotated as proline-rich protein, dehydration-responsive element-binding protein, leucine-rich repeat receptor protein kinase, bidirectional sugar transporter SWEET14, *etc* (Table 3).

Pathway analysis of DEGs

删除: **using KEGG and MapMan**

KEGG pathway analysis was performed on the DEGs in response to *S. sclerotiorum* infection in DS (Fig. S1) and DR (Fig. S2) genotypes. The plant-pathogen interaction was the top up-regulated pathway for both genotypes. In this pathway, 24 and 23 receptor-like proteins, which could be considered as PRRs, were up-regulated significantly by *S. sclerotiorum* infection in DS and DR genotypes, respectively (Table 4). Of these, 13 were uniquely up-regulated in DR genotype and its expression exhibited non-significantly changed in DS genotype, such as EIX1 and EIX2 homolog genes.

删除: both

删除: (Fig. S2)

By using MapMan software, a pathway was shown which consisting of 1089 and 886 DEGs by *S. sclerotiorum* infection in DS and DR genotypes (Fig. 3). In both genotypes, most of genes

删除: analysis

删除: 4

删除: A

involved in effector receptor (NLR), protein kinase MAPK, calcium-dependent signaling (*e.g.* CDPKs, CBLs and CMLs), transcription factor (*e.g.* ERF, WRKY and MYB), systemic acquired resistance (*e.g.* regulatory protein CBP60/SARD, disease resistance mediator MLO2/6/12, aminotransferase ALD1, pipecolate N-hydroxylase FMO1 and pipecolate oxidase SOX) and secondary metabolism (*e.g.* mevalonate pathway, methylerythritol phosphate pathway, p-coumaroyl-CoA biosynthesis and flavonoid biosynthesis) were up-regulated, whilst most of genes for proteolysis (*e.g.* cysteine-type peptidase, serine-type peptidase, aspartic-type peptidase and metallopeptidase activities) were down-regulated by *S. sclerotiorum* infection. Of above pathways, the number of up- or down-regulated genes in DS genotype was more than in DR genotype. However, a large number DEGs were also divided into hormone signaling, cell wall organisation and redox homeostasis, which were chosen to conduct a deeper evaluation of *S. sclerotiorum* resistant traits.

删除: *SAR,*

Plant hormone signaling pathways

By *S. sclerotiorum* infection, most of genes related to auxin signaling pathways were significantly down-regulated, and the number of these down-regulated genes were more in DS genotype than in DR genotype (S13 Table). However, most of genes related to signaling pathways of ethene (ET) and brassinolide (BR), were significantly up-regulated. The DS genotype had more up-regulated genes than the DR genotype in above pathways apart from ET signaling. The ET signaling pathways showed more up-regulated genes in DR genotype (11) compared to DS genotype (7), which was attributed to more uniquely up-regulated genes for receptor protein (ETR/ERS) and EIN3 transcription factor activity (Fig. 4A). After *S. sclerotiorum* infection, only the gene LOC110884479 encoding EIN3 transcription factor activity showed higher expression level in DR genotype than in DS genotype.

删除: 9

删除: 45

删除: B

Redox homeostasis

Redox homeostasis was affected by *S. sclerotiorum* infection, in which 94 DEGs in DS genotype and 68 in DR genotype. Most of genes for NADPH respiratory burst oxidase homolog involved in the ROS generation were up-regulated in both genotypes (S14 Table). In ROS scavenging, the DR genotype showed more up-regulated genes associated with SODs than the DS genotype (Fig. 4B). On the contrary, the DS genotype had more down-regulated genes involved in ascorbate-based redox regulation including APX and MDAR than the DR genotype. After *S. sclerotiorum* infection,

删除: (RBOHD/F)

删除: 0

删除: 65

删除: C

删除: more

the DR genotype showed six up-regulated and four down-regulated genes as compared to DS genotype.

Cell wall organisation

删除:

In cell wall organisation, 285 and 262 DEGs were identified by *S. sclerotiorum* infection in DS and DR lines, respectively. Most of genes involved in the cellulose synthase complex, xylan biosynthesis, heteromannan biosynthesis, and cutin polyester biosynthesis were down-regulated in both genotypes, while monolignol biosynthesis and expansin activities were up-regulated (S15 Table). However, we found several up-regulated genes related to expansin activities, such as alpha-class expansin, in which more in DR genotype compared to DS genotype (Fig. 4C). In addition, genes involved in pectin modification and degradation, such as pectate lyase showed a peculiar behaviour, most of them were up-regulated in DR genotype rather than DS genotype (Fig. 4E). After *S. sclerotiorum* infection, four genes LOC110871669, LOC110873721, LOC110904631 and LOC110941814 encoding pectate lyase showed higher expression level in DR genotype than in DS genotype.

删除: 1

删除: 45

删除: D

Validating the reliability of RNA-seq data by qRT-PCR

删除:

Nine genes were selected to validate the reliability of RNA-seq data by using qRT-PCR. The expression trends of selected genes obtained by qRT-PCR were good agreement with those measured by RNA-seq (Fig. 5). Of these, the CDPK9 (LOC110873588) and MAPK5 (LOC110935435) were significantly up-regulated by *S. sclerotiorum* infection in both genotypes (Fig. 5A and B). The CML23 (LOC110869082) and five NLRs (LOC110915177, LOC110877402, LOC110923780, LOC110883529 and LOC110929730) were uniquely up-regulated in the DR genotype and their expression but showed non-significantly changed in DS genotype (Fig. 5C-H). Notably, the LOC110935776 encoding NLR exhibited a the opposite expression pattern, which was down-regulated in DS genotype but dramatically up-regulated in the DR genotype (Fig. 5I).

Discussion

Differences in *S. sclerotiorum* resistance between the two sunflower genotypes

In this study, the DR genotype C6 displayed symptoms of *S. sclerotiorum* infection later (72 hpi) and with less severity after inoculation than DS genotype B728 (24 hpi), which was also reported by previous studies in regard to the defence response to *S. sclerotiorum* in other crop plants [30,31]. The assessments of visible lesions and hyphae further demonstrated the resistance difference between these two genotypes. We sampled the inoculated and mock-inoculated leaves at 24 hpi for RNA-seq analysis, when the slight phenotypic difference has just occurred. At this time point, the DS genotype had larger number of DEGs than the DR genotype, indicating that it is relatively hyper-responsive or more sensitive to *S. sclerotiorum* at the molecular level. The results were inconsistent with the results of previous studies in B. *napus*[30,32].

删除: 29

删除: 0

删除: 29

删除: 31

Expansin and pectate lyase activities contribute to resistance against *S. sclerotiorum*

We identified more down-regulated genes involved in chloroplast as well as photosynthesis in DS genotype compared with those in the DR genotype. Disruption of chloroplast function often resulted in the differentially expressed of genes involved in cell wall synthesis and modification. Cell wall, as the first defensive barrier against pathogens, undergoes dynamic remodeling to prevent infection by pathogens [33]. During the interaction with its host, *S. sclerotiorum* synthesizes and secretes oxalic acid and cell-wall-degrading enzymes, which could decrease cell wall rigidity, cause rapid cell collapse, and finally destroy expanding parts of infected tissue [34]. The extracellular enzymes produced by *S. sclerotiorum* can physically degrade plant cell wall components and consequently provide nutrients to drive the infection process [35]. Up-regulation of cellulose synthases, xyloglucan endotransglucosylase/hydrolase, monolignol biosynthesis, lignin deposition could reinforce the cell wall to react faster and stronger to subsequent *S. sclerotiorum* attack [36-38]. In this study, most of genes related to monolignol biosynthesis and expansin activities were up-regulated by *S. sclerotiorum* infection in both genotypes. Importantly, we found more up-regulated genes related to expansin activities in DR genotype compared to DS

删除: 2

删除: OA

删除: CWDE

删除: 3

删除: 4

删除: 5

删除: 7

genotype. Plant expansins as cell wall protein play a key role in many processes modify the cell wall, including relaxation and extension [39], and constitute a regular component against pathogen infection [40]. Overexpression of *EXLB8*, one of subfamilies of plant expansins, improved resistance to *S. sclerotiorum* in tobacco through physically modifying the cell wall structure as well as activating the phytohormone signaling pathways, such as JA-ET cooperation [41].

删除: 8
删除: 39
删除: 0

In addition, we found that the genes for pectate lyase were up-regulated in DR genotype rather than DS genotype by *S. sclerotiorum* infection. The homogalacturonan of pectin that could expose the polysaccharides at the early stage of invasion, was depolymerized by pectate lyase, which was released from pathogens [42-44]. By the degradation of polysaccharides, the plant cell wall produce oligosaccharides, which can enhance disease resistance via promoting ROS burst, MAPK activation and thus the expression of immune-responsive genes[45,46]. Our results suggested that the expansin and pectate lyase activities may be associated with *Sclerotinia* resistance in sunflower.

删除: 1
删除: 3
删除: 4
删除: 5

Differences in ROS scavenging between contrasting genotypes

The ROS are secondary messengers in the signal transduction process and play crucial roles in stress response and plant growth [47]. However, if the stress is too severe, excess ROS will cause programmed cell death and trigger host cell necrosis, which can promote the growth of necrotrophic pathogens such as *S. sclerotiorum* [48]. Organisms have complex antioxidant systems that regulate the balance between ROS production and ROS scavenging to combat oxidative stress caused by excess ROS [49]. SOD is involved in inactivating superoxide anions, which constitutes the front-line defense against ROS [50]. In this study, the increase of SOD activity was found only in the DR genotype after *S. sclerotiorum* infection. Most of genes associated with SOD were up-regulated in the DR genotype, indicated an elevated levels of antioxidant defence decreased oxidative damage and cell death in this genotype. Moreover, the pathway of ascorbate-glutathione cycle is essential for ROS scavenging, which can protect ROS signalling and avoid H_2O_2 toxicity [51,52]. We found higher activity of GST, APX and MDAR in DR genotype than in DS genotype after *S. sclerotiorum* infection. GSTs play a crucial role as cellular protectants in detoxification against oxidative damage, and were induced by *S.*

删除: 6
删除: PCD
删除: 7
删除: 8
删除: 49
删除: 0
删除: 1

sclerotiorum in this study, which has been also reported in the previous studies [53]. However, the DS genotype had more down-regulated genes associated with APX and MDAR involved in ascorbate-based redox regulation. APX as one of the most important peroxidases catalyzes reduction of H_2O_2 by the reducing power of ascorbate [54], which is the most important reducing substrate for H_2O_2 detoxification in plant cells [55]. MDAR can directly catalyses the regeneration of ascorbate at both plasmalemma and thylakoid membrane [56]. During the detoxification of ROS, Additionally, MDAR is capable of converting phenoxyl radicals into their respective parental phenols, which are very efficient antioxidants comparable to ascorbate [57]. Decreased activity of APX and MDAR only in DS leaves likely contributes to the cellular degradation before fungal hyphae, thereby increasing nutrient availability after OA-induced cell death [58].

Immune response to *S. sclerotiorum* in sunflower

The initial stage of PTI involves PRR stimulation [58]. A total of 57 genes for receptor-like kinases (RLKs) were as well-characterized PRR, which were up-regulated in *Brassica napus* by *S. sclerotiorum* infection [30]. A PRR gene, *ZmWAK*, was identified in maize by QTL mapping, which was up-regulated after infected and confered quantitative resistance to head smut [57]. In this study, 24 and 23 genes, in the DR and DS genotype, respectively, were identified as PRRs in the plant-pathogen interaction pathway, which were up-regulated by *S. sclerotiorum* infection. Importantly, we found 13 PRRs were uniquely up-regulated in the DR genotype, which might have a critical role in recognizing pathogen-associated molecular patterns induced by S. sclerotiorum and initiating the immune response.

Defense responses also begin with the intracellular recognition of the pathogen effector molecules through a series of plant R gene products, which leads to ETI [58]. In disease resistance breeding, ETI as an important trait is widely used to resist the biotrophic and hemibiotrophic instead of necrotrophic pathogens [59]. However, in *Arabidopsis*, RLM3 has been identified as R gene, which encodes a TIR domain known to affect broad-range immunity to several necrotrophic pathogens [60]. It is unknown whether sunflower has any R-genes may refered to the *S. sclerotiorum* resistance. In this study, we identified six R genes encoding NLR proteins by qRT-PCR, which were uniquely up-regulated in the DR genotype. Of these, the gene

删除: 2
删除: 3
删除: 4
删除: 4
删除: 5
删除: 6
删除: 7
删除: 29
删除: 6
删除: PAMP
删除: 7
删除: 8
删除: 59

LOC110935776 was down-regulated in DS genotype but dramatically up-regulated in DR genotype. In sunflower, it will be necessary to investigate whether these NLR genes play a role in the immune response to *S. sclerotiorum*.

Defense signaling pathways in response to *S. sclerotiorum* challenge

When recognizing invading pathogens, plants respond to pathogen attack by activation a large number of defense responses, in which a central role is signaling, including MAPK kinases, calcium-dependent signaling, plant hormone and transcription factors. Most of genes associated with MAPK kinases, CDPKs, CMLs, ET, BR, ERF, WRKY and MYB were up-regulated by *S. sclerotiorum* infection in this study. During *S. sclerotiorum* infection, MAPK is an important serine/threonine protein kinase that transduces signals from cytoplasm to nucleus [61]. The changes of transcription factors dependent on a series of phosphorylation was due to the MAPK cascade signalling pathway, which were activated after PRR stimulation [62,63]. The transcription factors MPK4 and the WRKY33 were demonstrated to assoiated with the resistance to *Botrytis cinerea* and *S. sclerotiorum* [64-66]. Over-expression of BnMAPK4 enhances resistance to *S. sclerotiorum* in transgenic *Brassica napus* [67].

In addition, the plant respond to *S. sclerotiorum* attack was also depend on the calcium (Ca) signaling pathway, which mainly included CDPKs and CMLs. It is a well known that the H_2O_2 accumulation can be positively regulated by CDPKs via phosphorylating in the plasma membrane and cytosol, and thus activate RBOHD/F [68]. These results suggest that these genes as the regulator might play a key role in cellular signaling networks to induce *S. sclerotinia* stress resistance. In this study, one gene CML23 were identified as uniquely up-regulation in the DR rather than in DS genotype.

Jasmonic acid (JA)/ET and SA-mediated signaling pathways are known to be major components of plant defense networks [69,70], which can act independently, synergistically, or in some cases antagonistically between host/pathogens [71]. In this study, genes associated with ET signal pathways were induced, whilst no salicylic acid (SA) and JA responsive genes were identified. ET could induce the pathogenesis related proteins and synthesize the phytoalexin against invading microbial pathogens [72]. Previous study showed that the ET responsive

删除: the
删除: tion
删除: of
删除: and
删除: the
删除: during this process
删除: played by
删除: 0
删除: relying
删除: 1
删除: 2
删除: 3
删除: 5
删除: 6
删除: 7
删除: 8
删除: 69
删除: 0
删除: PR
删除: 1

signaling seemed to proceed at a late stage of disease in the tolerant *B. carinata* but no such increase was found in the susceptible ones at any of the investigated time points [73]. We also found that the DR genotype had more up-regulated genes associated with ETR/ERS and transcription factor EIN3 in the ET signaling pathways compared to DS genotype. Our results suggested that the increase of ET signal transduction could contribute to resistance against *S. sclerotiorum*. Oppositely, more up-regulated genes for ET biosynthesis were found in DS genotype than in DR genotype (S13 Table). ET has been demonstrated to play a key role in the programmed cell death regulation [74], which might help the necrotrophic pathogen to absorb nutrients for continuded growth and development [70]. Thus, we proposed that blocking ET biosynthesis may alleviate *S. sclerotinia* in sunflower.

删除: 2

删除: 9

删除: PCD

删除: 3

删除: 69

Conclusions

RNA-Seq analysis was carried out in this study to analyze transcriptomic profiles of two sunflower genotypes viz., DS and DR that were systemically infected with *S. sclerotinia* and elucidate the molecular basis of the *S. sclerotiorum* resistance mechanism. The DEGs analysis showed that the DR genotype is less affected by *S. sclerotiorum* infection as compared to the DS genotype. Taken together the results from the GO, KEGG and MapMan analysis, a model was proposed to show the major molecular mechanisms underlying the defense responses against *S. sclerotiorum* in sunflower (Fig. 6). The oxalic acid and cell-wall-degrading enzymes as well as the excess of ROS, generated by the interaction between host and *S. sclerotiorum*, cause the programmed cell death, which help the *S. sclerotiorum* to obtain nutrients for growth and development. In the DR genotype, the expansins were up-regulated to restrain cell-wall-degrading enzymes; pectate lyase activities were up-regulated to activate the defense response; the SOD was up-regulated to eliminate of ROS; the ET signaling were up-regulated to activate the systemic acquired resistance and secondary metabolisms via a series of signal transduction. In DS genotype, however, the up-regulated of ET biosynthesis and the down-regulated of APX and MDAR facilitated the programmed cell death. Our results revealed how the cell wall modifying enzymes, antioxidants, hormone as well as signaling increased *S. sclerotiorum* resistance in DR relative to DS sunflower genotype. Overall, these finding will serve as a resource for further studies of the molecular mechanisms associated the *S. sclerotiorum* resistance in sunflower and development of

删除: OA

删除: CWDE

删除: PCD

删除: CWDE

删除: SAR

删除: PCD

effective strategies in *Sclerotinia*-resistance breeding.

Availability of data and materials

The data sets supporting the results of this article are included within the article and its additional files. All the raw data of the transcriptome sequence have been deposited in the NCBI BioProject database (https://www.ncbi.nlm.nih.gov/bioproject/PRJNA908908) under the BioProject PRJNA908908.

删除: The data discussed in this publication have been deposited in NCBI's Gene Expression Omnibus and are accessible through GEO Series accession number GSE220161 (https://www.ncbi.nlm.nih.gov/geo/query/acc.cgi?acc=GSE220161)

Author Contributions

Funding acquisition: Mingzhu Zhao, Liangji Cui, Bing Yi.

Methodology: Mingzhu Zhao, Bing Yi

Investigation: Mingzhu Zhao, Xiaohong Liu, Dexing Wang, Dianxiu Song, Enyu Sun.

Supervision: Jingang Liu, Liangshan Feng

Writing-original draft: Mingzhu Zhao.

Writing-review & editing: Jingang Liu, Liangshan Feng

Competing interests

删除: **Funding**

The study was supported by National Natural Science Foundation of China (32372211), China Agriculture Research System of MOF and MARA (CARS-14), and Shenyang Seed Industry Innovation Project (22-318-2-19), and Presidential Foundation of the Liaoning Academy of Agricultural Sciences (2023MS0505 and 2024YQ0403). The funders had no role in study design, data collection and analysis, decision to publish, or preparation of the manuscript.

We hereby confirm that all authors have declared that no competing interests exist.

Acknowledgment

We thank Prof. Wenjing Zheng, Institute of Rice Research, Liaoning Academy of Agricultural Sciences, to review the manuscript.

Figure Captions

Fig. 1 Visible lesions of leaves at 24 hpi (A), 48 hpi (B) and 72 hpi (C) and chlorosis phenomenon (D and E) and mycelial growth (F and G) of two genotypes at 24 hpi by electron microscopy. Fungal hyphae shows green fluorescence, cell walls of plant show red fluorescence (F and G) of two genotypes at 24 hpi by Leaf Fungal Hyphae WGA-PI staining.

Fig. 2 Differentially expressed genes (DEGs) in each pairwise comparison. (A) Volcano plot. The x-axis and y-axis represents the log2 (fold change) and −log10 (FDR-value). The red dots represent up-regulated genes with $\log_2$ Fold_change >1 and FDR <0.01 and the green dots represent down-regulated genes with $\log_2$ Fold_change < −1 and FDR <0.01. (B) Based on the two comparisons performed, two Venn diagrams showed the numbers of genes that are up- and

down-regulated. Overlapping circles represent genes up- or down-regulated in both comparisons.

Fig. 3 Infection with *S. sclerotiorum* results in DEGs at the DS (A) and DR (B) genotypes as visualized in MapMan. Red and green indicate DEGs that are up-regulated and down-regulated, respectively.

Fig. 4 Heat map of DEGs related to ET signaling (A), ROS (B), expansin (C) and pectin (D). The gene expression levels were compared between *S. sclerotiorum*- and mock-inoculated samples of the same genotype as well as between the DR and DS genotype under the same conditions.

Fig. 5 The expression pattern of nine genes were verified by qRT-PCR.

Fig. 6 Sunflower defense responses against *S. sclerotiorum* described in a molecular model. CWDE: cell wall-degrading enzymes; OA: oxalic acid; TF: transcription factor; MAPK- mitogen activated protein kinase.

Supporting Information

S1 Table. The gene-specific primers for qRT-PCR.

S2 Table. 119 constitutive expressed genes are induced by *S. sclerotiorum* infection in the DS genotype and are already expressed in the DR genotype in mock-inoculated control.

S3 Table. GO analysis to identify enriched gene ontologies among the up-regulated DEGs in DS genotype B728.

S4 Table. GO analysis to identify enriched gene ontologies among the down-regulated DEGs in DS genotype B728.

S5 Table. GO analysis to identify enriched gene ontologies among the up-regulated DEGs in DR genotype C6.

S6 Table. GO analysis to identify enriched gene ontologies among the down-regulated DEGs in DR genotype C6.

S7 Table. GO analysis to identify enriched gene ontologies among the common up-regulated DEGs in DS and DR genotypes.

S8 Table. GO analysis to identify enriched gene ontologies among the common up-regulated DEGs in DS and DR genotypes.

S9 Table. GO analysis to identify enriched gene ontologies among the unique up-regulated DEGs in DS genotype B728.

S10 Table. GO analysis to identify enriched gene ontologies among the unique down-regulated DEGs in DS genotype B728.

S11 Table. GO analysis to identify enriched gene ontologies among the unique up-regulated DEGs in DT genotype C6.

S12 Table. GO analysis to identify enriched gene ontologies among the unique down-regulated DEGs in DT genotype C6.

S13 Table. DEGs involved in phytohormone action by MapMan analysis.

S14 Table. DEGs involved in redox homeostasis by MapMan analysis.

S15 Table. DEGs involved in cell wall organisation by MapMan analysis.

S1 Fig. Kyoto Encyclopedia of Genes and Genome (KEGG) analysis for up- (A) and down-regulated (B) DEGs in DS genotype, respectively.

S2 Fig. Kyoto Encyclopedia of Genes and Genome (KEGG) analysis for up- (A) and down-regulated (B) DEGs in DR genotype, respectively.

删除: **Fig. 3** Twenty common GO terms in heat map identified from the up- (A) and down-regulated (B) genes in both the genotypes in response to *S. sclerotiorum*.

删除: **4**

删除: (A) and the heat map of genes related to ET signaling (B), ROS (C), expansin (D) and pectin (E).

删除: T

删除: T

删除: T

删除: S7 Table. GO analysis to identify enriched gene ontologies among the unique up-regulated DEGs in DT genotype C6.

删除: GO analysis to identify enriched gene ontologies among the unique down-regulated DEGs in DT genotype C6.

删除: **9**

删除: **0**

删除: **1**

删除: Gene Ontology (GO) based pathway analysis for up- and down-regulated DEGs in DR and DS genotypes, respectively.
S2 Fig.

删除: DR and

删除: s

REFERENCES

1.Liu J, Zhang Y, Meng Q, Shi F, Ma L, Li Y. (2017) Physiological and biochemical responses in sunflower leaves infected by *Sclerotinia sclerotiorum*. Physiol Mol Plant P 100:41-48.

2.Vuong TD, Hoffman DD, Diers BW, Miller JF, Steadman JR, Hartman GL. (2004) Evaluation of soybean, dry bean, and sunflower for resistance to *Sclerotinia sclerotiorum*. Crop Sci. 44(3): 777-783.

3.Joshi RK, Megha S, Basu U, Rahman MH, Kav NN. (2016) Genome wide identification and functional prediction of long non-coding RNAs responsive to *Sclerotinia sclerotiorum* infection in *Brassica napus*. PLoS ONE 11: e0158784

4.Prats E, Galindo JC, Bazzalo ME, León A, Macías FA, Rubiales D, et al. (2007) Antifungal activity of a new phenolic compound from capitulum of a head rot-resistant sunflower genotype. J Chem Ecol. 33: 2245-2253.

5.Lu G. (2003) Engineering *Sclerotinia sclerotiorum* resistance in oilseed crops. Arf J Biotechnol. 2:509-516.

6.Clarkson JP, Fawcett L, Anthony SG, Young C. (2014) A model for *Sclerotinia sclerotiorum* infection and disease development in lettuce, based on the effects of temperature, relative humidity and ascospore density. PLoS ONE 9: e94049.

7.Bolton MD, Thomma BP, Nelson BD. (2006) *Sclerotinia sclerotiorum* (Lib.) de Bary: biology and molecular traits of a cosmopolitan pathogen. Mol Plant Pathol 7, 1-16.

8.del Río LE, Bradley CA, Henson RA, Endres GJ, Hanson BK, McKay K, et al. (2007) Impact of *Sclerotinia* stem rot on yield of canola. Plant Dis 91: 191-194.

9.Zhao J, Peltier AJ, Meng J, Osborn TC, Grau CR. (2004) Evaluation of *Sclerotinia* stem rot resistance in oilseed *Brassica napus* using a petiole inoculation technique under greenhouse conditions. Plant Dis 88:1033-1039.

10.Bastien M, Sonah H, Belzile F. (2014) Genome wide association mapping of *Sclerotinia sclerotiorum* resistance in soybean with a genotyping-by-sequencing approach. Plant Genome-US 7:2013-10.

11.Jones JD, Dang JL. (2006) The plant immune system. Nature 444:323-329.

12.Tena G. (2021) PTI and ETI are one. Nat. Plants 7:1527-1527.

13.Gao X, Dang X, Yan F, Li Y, Xu J, Tian S, et al. (2022) ANGUSTIFOLIA negatively regulates resistance to *Sclerotinia sclerotiorum* via modulation of PTI and JA signalling pathways in *Arabidopsis thaliana*. Mol Plant Pathol 23:1091-1106.

14.Chisholm ST, Coaker G, Day B, Staskawicz BJ. (2006) Host-microbe interactions: shaping the evolution of the plant immune response. Cell 124: 803-814.

15.Badet T, Léger O, Barascud M, Voisin D, Sadon P, Vincent R, et al. (2019) Expression polymorphism at the ARPC 4 locus links the actin cytoskeleton with quantitative disease resistance to *Sclerotinia sclerotiorum* in *Arabidopsis thaliana*. New Phytol 222, 480-496.

16.Wen L. (2013) Cell death in plant immune response to necrotrophs. J Plant Biochem Physiol 1: 1-3.

17.Wang JQ, Mao L. (2019) The ERK pathway: molecular mechanisms and treatment of depression. Mol Neurobiol 56:6197-6205.

18.Yang G, Tang L, Gong Y, Xie J, Fu Y, Jiang D, et al. (2018) A cerato-platanin protein SsCP1 targets plant PR1 and contributes to virulence of *Sclerotinia sclerotiorum*. New Phytol 217:739-755.

19.Fass MI, Rivarola M, Ehrenbolger GF, Maringolo CA, Montecchia JF, Quiroz F, et al. (2020) Exploring sunflower responses to *Sclerotinia* head rot at early stages of infection using RNA-seq analysis. Sci Rep-UK 10:1-14.

20.Joshi RK, Megha S, Rahman MH, Basu U, Kav NN. (2016) A global study of transcriptome dynamics in canola (*Brassica napus* L.) responsive to *Sclerotinia sclerotiorum* infection using RNA-Seq Gene 590: 57-67.

21.Girard IJ, Tong C, Becker MG, Mao X, Huang J, de Kievit T, et al. (2017) RNA sequencing of *Brassica napus* reveals cellular redox control of *Sclerotinia* infection. J Exp Bot 68:5079-5091.

22.Ghareeb H, Becker A, Iven T, Feussner I, Schirawski J. (2011) Sporisorium reilianum infection changes inflorescence and branching architectures of maize. Plant Physiol 156: 2037-2052.

23.Giannopolitis CN, Ries SK. (1977) Superoxide dismutases: II. Purification and quantitative relationship with water-soluble protein in seedlings. Plant Physiol 59(2):315-318.

24.Shannon LM, Kay E, Lew JY. (1996) Peroxidase isozymes from horseradish roots I. Isolation and physical properties. J Biol Chem 241:2166–2172.

25.Aebi H. (1984) Catalase in vitro. Method Enzymol 105:121–126.

26.Mauch F, Dudler R. (1993) Differential induction of distinct glutathioneS-transferases of Wheat by xenobiotics and by pathogen attack. Plant Physiol 102:1193–1201.

27.Nakano Y, Asada K. (1981) Hydrogen peroxide is scavenged by ascorbate-specific peroxidases in spinach chloroplasts. Plant Cell Physiol 22: 867–880.

28.Drazkiewicz M, Skorzy nska-Polit E, Krupa Z. (2003) Response of the ascorbate-glutathione cycle to excess copper in *Arabidopsis thaliana* (L.). Plant Sci 164: 95–202.

29.Badouin H, Gouzy J, Grassa CJ, Murat F, Staton SE, Cottret L, et al. (2017). The sunflower genome provides insights into oil metabolism, flowering and Asterid evolution. Nature 546(7656): 148–152.

30.Wu J, Zhao Q, Yang Q, Liu H, Li Q, Yi X, et al. (2016) Comparative transcriptomic analysis uncovers the complex genetic network for resistance to *Sclerotinia sclerotiorum* in *Brassica napus*. SCI Rep-UK 6:1-16.

删除: 29

31.Mwape VW, Mobegi FM, Regmi R, Newman TE, Kamphuis LG, Derbyshire MC. (2021) Analysis of differentially expressed *Sclerotinia sclerotiorum* genes during the interaction with moderately resistant and highly susceptible chickpea lines. BMC Genomics 22:1-14.

删除: 0

32.Chittem K, Yajima WR, Goswami RS, del Río Mendoza LE. (2020) Transcriptome analysis of the plant pathogen *Sclerotinia sclerotiorum* interaction with resistant and susceptible canola (*Brassica napus*) lines. PLoS ONE 15: e0229844.

删除: 1

33.Cosgrove DJ. (2005) Growth of the plant cell wall. Nat Rev Mol Cell Bio 6: 850-861.

删除: 2

34.Riou C, Hervé C, Pacquit V, Dabos P, Lescure B. (2002) Expression of an *Arabidopsis* lectin kinase receptor gene, lecRK-a1, is induced during senescence, wounding and in response to oligogalacturonic acids. Plant Physiol Bioch 40:431-438.

删除: 3

35.Zhao Y, Song D, Sun J, Li L. (2013) Populus endo-beta-mannanase PtrMAN6 plays a role in

删除: 4

coordinating cell wall remodeling with suppression of secondary wall thickening through generation of oligosaccharide signals. Plant J 74:473-485.

36. Wei W, Xiong Y, Zhu W, Wang N, Yang G, Peng F. (2016) Colletotrichum higginsianum mitogen-activated protein kinase ChMK1: role in growth, cell wall integrity, colony melanization, and pathogenicity. Front Microbiol 7:1212. 删除: 5

37. Madany MM, Saleh AM, Habeeb TH, Hozzei WN, AbdElgawad H. (2020) Silicon dioxide nanoparticles alleviate the threats of broomrape infection in tomato by inducing cell wall fortification and modulating ROS homeostasis. Environ Sci-NANO 7: 1415-1430. 删除: 6

38. Eynck C, Séguin-Swartz G, Clarke WE, Parkin IA. (2014) Monolignol biosynthesis is associated with resistance to *Sclerotinia sclerotiorum* in Camelina sativa. Mol Plant Pathol. 52;13: 887-899. 删除: 7 删除: INETTE

39. Cosgrove C. (2015) Comparing aquatic invertebrate communities on pin oak, cottonwood, and red maple leaf litter in vernal pools in northeastern Ohio. Kent State University. 删除: 8

40. Marowa P, Ding A, Kong Y. (2016) Expansins: roles in plant growth and potential applications in crop improvement. Plant Cell Rep 35;949-965. 删除: 39

41. Brasileiro AC, Lacorte C, Pereira BM, Oliveira TN, Ferreira DS, Mota AP, et al. (2021) Ectopic expression of an expansin-like B gene from wild Arachis enhances resistance to both abiotic and biotic stresses. Plant J 107:1681-1696. 删除: 0

42. Lombard V, Bernard T, Rancurel C, Brumer H, Coutinho PM, Henrissat B. (2010) A hierarchical classification of polysaccharide lyases for glycogenomics. Biochem J 432: 437-444. 删除: 1

43. Kubicek CP, Starr TL, Glass NL. (2014) Plant cell wall-degrading enzymes and their secretion in plant-pathogenic fungi. Annu Rev Phytopathol 52:427-451. 删除: 2

44. Yakoby N, Kobiler I, Dinoor A, Prusky D. (2000) pH regulation of pectate lyase secretion modulates the attack of Colletotrichum gloeosporioides on avocado fruits. Appl Environ Microb 66, 1026-1030. 删除: 3

45. Yang H, Yang Y L, Li G Q, Yu Q, Yang J. (2021) Identifications of immune-responsive genes for adaptative traits by comparative transcriptome analysis of spleen tissue from Kazakh and Suffolk sheep. Sci Rep-UK 11:1-16. 删除: 4

46. Wan J, He M, Hou Q, Zou L, Yang Y, Wei Y, Chen X. (2021) Cell wall associated immunity in plants. Stress 1:1-15. 删除: 5

47. Mittler R, Vanderauwera S, Suzuki N, Miller G A D, Tognetti V B, Vandepoele K, et al. (2011) ROS signaling: the new wave?. Trends Plant Sci 16: 300-309. 删除: 6

48. Apel K, Hirt H. (2004) Reactive oxygen species: metabolism, oxidative stress, and signaling transduction. Annu Rev Plant Biol 55:373. 删除: 7

49. Ding Y, Mei J, Chai Y, Yang W, Mao Y, Yan B, et al. (2020) *Sclerotinia sclerotiorum* utilizes host-derived copper for ROS detoxification and infection. Plos Pathog 16: e1008919. 删除: 8

50. Bowler C, Montagu M, Inze D. (1992) Superoxide dismutase and stress resistance. Annu Rev Plant Biol 43(1): 83-116. 删除: 49

51. Foyer CH, Noctor G. (2011) Ascorbate and glutathione: the heart of the redox hub. Plant Physiol 155: 2-18. 删除: 0

52. de Pinto MC, Tommasi F, De Gara L. (2002) Changes in the antioxidant systems as part of the signaling pathway responsible for the programmed cell death activated by nitric oxide and reactive oxygen species in tobacco Bright-Yellow 2 cells. Plant Physiol 130: 698-708. 删除: 1

53.Zhao J, Buchwaldt L, Rimmer SR, Sharpe A, McGregor L, Bekkaoui D, et al. (2009) Patterns of differential gene expression in Brassica napus cultivars infected with *Sclerotinia sclerotiorum*. Mol Plant Pathol 10:635-649.

54.Jain N, Menache I, Naor JS, Shepherd FB. (2012) Topology-aware vm migration in bandwidth oversubscribed datacenter networks. In International Colloquium on Automata, Languages, and Programming, Springer, Berlin, Heidelberg 586-597.

55.Noctor G, Foyer CH. (1998) Ascorbate and glutathione: keeping active oxygen under control. Annu Rev Plant Biol 49: 249-279.

56.Sakihama Y, Mano JI, Sano S, Asada K, Yamasaki H. (2000) Reduction of phenoxyl radicals mediated by monodehydroascorbate reductase. Biochem Bioph Res CO 279:949-954.

57.Kim I, Xu W, Reed JC. (2008) Cell death and endoplasmic reticulum stress: disease relevance and therapeutic opportunities. Nat Rev Drug Discov 7:1013-1030.

58.Zuo J, Li J. Molecular dissection of complex agronomic traits of rice: a team effort by Chinese scientists in recent years. Nalt Sci Rev. 2014;1:253-276.

59.Jones JDG, Dangl JL. (2006) The plant immune system. Nature 444(7117): 323-329.

60.Mengiste T. (2012) Plant immunity to necrotrophs. Annu Rev Phytopathol 50: 267-29.

61.Sun Y, Wang C, Yang B, Wu F, Hao X, Liang W, Niu F, Yan J, Zhang H, Wang B, Deyholos MK, Jiang YQ. (2014) Identification and functional analysis of mitogen-activated protein kinase kinase kinase (MAPKKK) genes in canola (*Brassica napus* L.). J Exp Bot 65:2171-2188.

62.Asai T, Tena G, Plotnikova J, Willmann MR, Chiu WL, Gomez-Gomez L, et al. (2002) MAP kinase signalling cascade in Arabidopsis innate immunity. Nature 415:977-983.

63.Wang Z, Ma LY, Cao J, Li YL, Ding LN, Zhu KM, et al. (2019) Recent advances in mechanisms of plant defense to *Sclerotinia sclerotiorum*. Front plant Sci 10: 1314.

64.Wang Z, Mao H, Dong C, Ji R, Cai L, Fu H et al. (2009) Overexpression of *Brassica napus* MPK4 enhances resistance to *Sclerotinia sclerotiorum* in oilseed rape. Mol Plant Microbe In 22:235-244.

65.Wang Z, Fang H, Chen Y, Chen K, Li G, Gu S, et al. (2014) Overexpression of BnWRKY33 in oilseed rape enhances resistance to *Sclerotinia sclerotiorum*. Mol Plant Pathol 15: 677-689.

66.Wang C, Yao J, Du X, Zhang Y, Sun Y, Rollins JA et al. (2015) The Arabidopsis mediator complex subunit16 is a key component of basal resistance against the necrotrophic fungal pathogen *Sclerotinia sclerotiorum*. Plant Physiol 169:856-872.

67.Takahashi F, Mizoguchi T, Yoshida R, Ichimura K, Shinozaki K. (2011) Calmodulin-dependent activation of MAP kinase for ROS homeostasis in *Arabidopsis*. Mol Cell 41(6):649–660.

68.Liu J, He B, Chen Q, Li J, Xiong Q, Yue G, et al. (2016) Direct synthesis of hydrogen peroxide from plasma-water interactions. Sci Rep-UK 6: 1-7.

69.McDowell JM, Dang JL. (2000) Signal transduction in the plant immune response. Trends Biochem Sci 25:79–82.

70.Martinez C, Blanc F, Le Claire E, Besnard O, Nicole M, Baccou JC. (2001) Salicylic acid and ethylene pathways are differentially activated in melon cotyledons by active or heat-denatured cellulase from Trichoderma longibrachiatum. Plant Physiol 127: 334–344.

71.Glazebrook J. (2005) Contrasting mechanisms of defense against biotrophic and necrotrophic pathogens. Annu Rev Phytopathol 43:205–227.

72.Broekaert IJ, Walker WA. (2006) Probiotics and chronic disease. J. Clin Gastroenterol

40:270-274.

73. Yang B. (2009) Characterization of the *Brassica napus*-fungal pathogen interaction. Doctoral dissertation, University of Alberta.

删除: 2

74. Asai T, Stone JM, Heard JE, Kovtun Y, Yorgey P, Sheen J, et al. (2000) Fumonisin B1–induced cell death in *Arabidopsis* protoplasts requires jasmonate-, ethylene-, and salicylate-dependent signaling pathways. Plant Cell 12:1823-1835.

删除: 3

Table 1 Activity changes in antioxidant enzymes in DS (B728) and DR (C6) sunflower inbred lines at 24 hpi by S. sclerotiorum infection.

Genotype	Treatment	SOD activity (ul·mg^{-1} protein·min^{-1})	POD activity (mmol·mg^{-1} protein·min^{-1})	CAT activity (mmol·mg^{-1} protein·min^{-1})	GST activity (μmol·mg^{-1} protein·min^{-1})	APX activity (mmol·mg^{-1} protein·min^{-1})	MDAR activity (mmol·mg^{-1} protein·min^{-1})
B728	Control	0.95±0.11b	14.35±1.35d	10.32±1.16d	20.64±2.02d	0.95±0.12a	5.37±0.92b
	inoculated	1.14±0.12b	24.36±2.02b	25.34±2.33b	31.27±2.33b	0.68±0.13b	3.28±0.84c
C6	Control	1.02±0.11b	18.37±1.39c	13.87±2.01c	25.64±2.12c	0.98±0.09a	6.11±0.81a
	inoculated	2.15±0.15a	30.11±2.87a	29.72±2.56a	35.69±2.24a	1.02±0.12a	6.59±0.72a

Table 2 Top 20 uniquely up-regulated genes in the DR genotype

Gene	Log_2 FC		Annotation
	B728	C6	
LOC110895548	-	9.24	Protein PLANT CADMIUM RESISTANCE 2
LOC110929546	-	8.93	Expansin-like B1
LOC110918750	-	8.25	Lignin-forming anionic peroxidase
LOC110885989	-	8.21	1-aminocyclopropane-1-carboxylate oxidase 3
LOC110886982	-	7.85	Berberine bridge enzyme-like 13
LOC110922271	-	7.83	EP1-like glycoprotein 4
LOC110879630	-	7.79	Probable L-gulonolactone oxidase 6
LOC110935776	-1.62	7.78	TMV resistance protein N
LOC110871630	-	7.73	Regulator of G-protein signaling 1
LOC110940063	-	7.70	Dirigent protein 21
LOC110928163	-	7.53	Cytochrome P450 87A3
LOC110877038	-	7.43	Cytokinin dehydrogenase 3
LOC110888580	-	7.39	Probable carboxylesterase 18
LOC110901085	-	7.38	-
LOC110934894	-	7.32	Ethylene-responsive transcription factor ERF098
LOC110925525	-	7.29	Hydroxycinnamoyltransferase 4
LOC110909848	-	7.25	Short-chain dehydrogenase reductase 2a
LOC110899788	-	7.21	1-aminocyclopropane-1-carboxylate oxidase homolog 1
LOC110945103	-	7.13	Cytochrome P450 71AV8
LOC110900121	-	7.06	Hydroquinone glucosyltransferase

Table 3 Top 20 uniquely down-regulated genes in the DR genotype

Gene	Log_2 FC		Annotation
	B728	C6	
LOC110885111	-5.70	-3.21	Proline-rich protein 4
LOC110930392	-5.55	-	Early light-induced protein 2
LOC110891330	-5.40	-	Soyasapogenol B glucuronide galactosyltransferase
LOC110869264	-4.92	-	Dehydration-responsive element-binding protein 1D
LOC110921230	-4.85	-2.19	-
LOC110909326	-4.54	-	-
LOC110869165	-4.54	-	Cyclin-U4-1
LOC110915744	-4.54	-	Leucine-rich repeat receptor protein kinase HPCA1
LOC110941770	-4.49	-	Bidirectional sugar transporter SWEET14
LOC110895020	-4.40	0.33	Probable galacturonosyltransferase-like 9
LOC110917579	-4.37	-0.67	Protein PHOSPHATE-INDUCED 1
LOC110908033	-4.19	-2.38	UPF0481 protein At3g47200
LOC110928088	-4.15	-	Protein SRC2
LOC110873198	-4.09	-1.73	Transcription repressor OFP1

LOC110929642	-4.06	-1.37	Receptor-like protein kinase THESEUS 1
LOC110912169	-4.06	-3.72	Probable inactive receptor kinase At1g48480
LOC110931795	-4.04	-2.49	-
LOC110864816	-4.01	-	GDSL esterase/lipase At4g01130
LOC110929231	-3.98	-1.20	Calmodulin-binding receptor kinase CaMRLK
LOC110868988	-3.94	-2.02	Uncharacterized protein At5g39865

Table 4 A list of transmembrane recognition receptors (PRRs) involved in plant-pathogen interaction pathway by KEGG analysis

Gene	Log_2 FC		Annotation
	B728	C6	
LOC110867023	5.60	6.46	Receptor-like protein 12
LOC110883941	4.02	3.60	Receptor-like protein 12
LOC110905009	2.29	1.71	Receptor-like protein EIX1
LOC110943528	2.50	1.42	Receptor-like protein EIX1
LOC110907898	5.24	3.84	Receptor-like protein EIX1
LOC110867197	1.66	1.20	Receptor-like protein EIX2
LOC110894990	6.67	4.51	Receptor-like protein EIX2
LOC110901887	3.27	2.31	Receptor-like protein EIX2
LOC110884455	5.47	3.79	Receptor-like protein EIX2 isoform X1
LOC110904904	3.57	1.12	Receptor-like protein EIX2 isoform X1
LOC110911286	-1.60	-1.26	Receptor-like protein 12
LOC110927258	7.29	-	Receptor-like protein 12
LOC110880537	5.50	-	Receptor-like protein 12
LOC110898370	2.33	-	Receptor-like protein 12
LOC110899583	6.89	-	Receptor-like protein 32
LOC110932987	7.90	-	Receptor-like protein 37
LOC110911343	2.13	-	Receptor-like protein EIX1
LOC110935701	2.81	-	Receptor-like protein EIX1 precursor
LOC110883962	2.01	-	Receptor-like protein EIX2
LOC110899544	3.10	-	Receptor-like protein EIX2
LOC110932925	7.28	-	Receptor-like protein EIX2
LOC110932954	6.64	-	Receptor-like protein EIX2
LOC110909891	1.04	-	Receptor-like protein EIX2
LOC110883961	2.45	-	Receptor-like protein EIX2
LOC110877393	1.16	-	Receptor-like protein EIX2 isoform X1
LOC110873395	-1.82	-	Receptor-like protein EIX2
LOC110880683	-	2.01	Receptor-like protein 12
LOC110904906	-	1.07	Receptor-like protein 12
LOC110872328	-	1.03	Receptor-like protein 12
LOC110867178	-	1.33	Receptor-like protein EIX1
LOC110872190	-	1.72	Receptor-like protein EIX2

LOC110932995	-	1.43	Receptor-like protein EIX2
LOC110927229	-	1.11	Receptor-like protein EIX2
LOC110924352	-	1.79	Receptor-like protein EIX2
LOC110887861	-	1.55	Receptor-like protein EIX2
LOC110880528	-	3.40	Receptor-like protein EIX2 isoform X1
LOC110867209	-	1.85	Receptor-like protein EIX2 isoform X1
LOC110867212	-	1.40	Receptor-like protein EIX2 isoform X1
LOC110872808	-	1.09	Receptor-like protein EIX2 isoform X1

Reviewer #2:

Please add the NCBI BioProject ID 'PRJNA908908' in the manuscript.

Response: All the raw data of the transcriptome sequence have been deposited in the NCBI BioProject database (https://www.ncbi.nlm.nih.gov/bioproject/PRJNA908908) under the BioProject PRJNA908908.

According to figure 4 B-E (figure 6 in previous version), nearly most genes are differentially expressed in both DS and DR genotypes ($|log2(FC)| > 1$), although the fold change varied. The expression of these genes was not statistically compared between DS and DR genotypes to narrow down the candidates, which could have been inferred from this data.

Response: The expression of these genes was compared between DS and DR genotypes, which were shown in Fig. 4(A-D), and the results section.

The read cleaning method is unclear and irreproducible. For example, a read should not be 'annotated based on the reference genome'. This revised method section still contains many errors, making the results unreliable.

Response: Raw reads of fastq format were firstly processed through in-house perl scripts. In this step, the clean reads were obtained by removing adapter-containing reads, ploy-N-containing reads, and low quality reads from raw data. The Q20, Q30, GC-content and sequence duplication level of the clean data were calculated. All the downstream analyses were based on clean data with high quality. The adaptor sequences and low-quality sequence reads were removed from the data sets. Raw sequences were transformed into clean reads after data processing. These clean reads were then mapped to the reference genome sequence. Only reads with a perfect match or one mismatch were further analyzed and annotated based on the sunflower reference genome. The HanXRQr1.0 genome was used as reference in the present study. Then, Hisat2 tools soft were used to map with reference genome. The StringTie Reference Annotation Based Transcript (RABT) assembly method was used to construct and identify both known and novel transcripts from Hisat2 alignment results.

The reference genome information is still not provided, making it impossible to assess the results. Not only the 'new genes', but also the 'reference genes' have only an 'ID' available, which largely limits the contribution to the community.

Response: The HanXRQr1.0 genome was used as reference in the present study.

The numbers of expressed genes were previously 22,077 and 22,579, but in the present version, they increased a lot. Again, without the information of the reference genome, it is impossible to know what happened.

Response: After careful inspection again， in the present version, the numbers of expressed genes were false, and those in the previous version were correct.

Without sufficient data, I cannot comment on the numbers listed.

Reviewer #3:
Authors have conducted transcriptome analysis of a resistant and a susceptible genotype of sunflower against Sclerotinia sclerotiorum. I have some suggestions and comments, which are given below. Page numbers are based on the revised, track-change version.
Major comments:
1) Figure-4, S1, and S2 are not legible. Hence, I couldn't check the information associated with these figures. These figures need to be updated.
Response: These figures were modified.

2) One major criticism is that all the follow-up analyses were done with the DEGs (Differentially Expressed Genes) upon infection in both the resistant and the susceptible genotypes. This was pointed out by one of the reviewers. However, the author didn't do a good job of resolving the issue. Ideally, it would be better to focus on DEGs that are unique to either the resistant or the susceptible genotype. Alternatively, performing clustering of DEGs could have been better.
Response: In GO analysis, the unique up- or down-regulated DEGs in either DS genotype B728 or DR genotype C6.

3) English is very poor in some instances. For example, on page 17, lines 2-3, sentence construction is not correct. The same is true for line-8-9. There are multiple examples like this throughout the manuscript. Correcting those will improve the readability of the manuscript.
Response: According the suggestion, these sentences were modified.

4) Did you use any filtering to get rid of the low-expressed genes, otherwise there will be DEGs that were expressed barely.
Response: Genes with low expression (FPKM <1) will be eliminated by default in the analysis process.

Minor comments:
1) Usage of abbreviations should be kept at a minimum level. CWDE for 'cell wall degrading enzyme' (page 3, line-11) is unacceptable.
Response: Many abbreviations were deleted in the revised manuscript, such as for oxalic acid, cell-wall-degrading enzymes, programmed cell death, systemic acquired resistance, programmed cell death, pathogenesis related proteins, pathogen-associated molecular pattern.

2) Page-5, line-6, why 24 hpi was used for the experiment?
Response: As the visible lesions appeared in DS genotype rather than DR genotype at 24 h post-inoculation, then the 20 leaves samples within the range of 0.5 cm bordering the extending lesion from inoculated and mock-inoculated plants were harvested for each replicate.

3) Page 6, line 20, how the raw reads were processed?
Response: Raw reads of fastq format were firstly processed through in-house perl scripts. In this step, the clean reads were obtained by removing adapter-containing reads, ploy-N-containing reads, and low quality reads from raw data. The Q20, Q30, GC-content and sequence duplication level of the clean data were calculated. All the downstream analyses were based on clean data with

high quality.

4) What kind of analysis was performed using MapMan? MapMan is a software, not an analysis.
Response: According the suggestion, the MapMan analysis was modified as MapMan software.

5) Page 8, line 27, what are the criteria for being expressed?
Response: FPKM>1.

6) GO analysis of all DEGs is meaningless, authors should have focused on unique DEGs.
Response: The GO analysis of all DEGs was deleted in the revised manuscript.

7) Page-9, line-23, "In the CC category, "cell", "cell part"……were most frequent". Is this kind of information meaningful?
Response: The GO analysis of all DEGs was deleted in the revised manuscript.

8) Common GOs for both genotypes represented in Figure-3, are not very informative.
Response: The Figure-3 in previous version was deleted, and the S7-8 Tables were added in the present version. The S7-8 Tables were the GO analysis to identify enriched gene ontologies among the common DEGs in DS and DR genotypes.

9) Page 10, lines 11-18, describe GO terms associated with the unique DEGs for each genotype. This section is useful. Authors should expand this section.
Response: According the suggestion, the unique up- and down-regulated DEGs in DS genotype B728 were used for GO analysis, which were also shown in S9-10 Tables.

10) The result section for the analysis using MapMan is very rudimentary.
Response: According another reviewer's suggestion, in the pathway analysis of DEGs using MapMan, the expressions of these genes involved in plant hormone signaling pathways, redox homeostasis and cell wall organisation were compared between DS and DR genotypes, which were shown in Fig. 4(A-D), and the results section.
