# Supplementary material for: Comparative transcriptome analysis in two contrasting genotypes for Sclerotinia sclerotiorum resistance in sunflower
Source: PLoS One. 2024 Dec 19;19(12):e0315458. doi: 10.1371/journal.pone.0315458 (PMC11658501; doi:10.1371/journal.pone.0315458)
Supplement: Attachment — Submitted filename: ResponseComments.docx [file pone.0315458.s018.docx]

Dear editor,

Thank you very much for your letter and giving me the information of comments from the reviewers about the Manuscript (ID PONE-D-23-14214). We also thank to the reviewer for their suggestions, which would help me to improve the quality of the paper. According to the reviewer’s comments and your advice, the manuscript was revised. Our revision was highlighted in the revised manuscript with the Word tracked mode. Please tell us if the revised manuscript still had deficiencies.

Best Regards

Sincerely yours,

Jingang Liu

**Journal Requirements**

1. Please ensure that your manuscript meets PLOS ONE's style requirements, including those for file naming.

Answer: The manuscript was revised according to the PLOS ONE's style requirements.

2. Thank you for stating the following financial disclosure: The study was supported by China Agriculture Research System of MOF and MARA (CARS-14), and Shenyang Seed Industry Innovation Project (22-318-2-19). Please state what role the funders took in the study.

Answer: We have stated that "The funders had no role in study design, data collection and analysis, decision to publish, or preparation of the manuscript."

3. We note that you have stated that you will provide repository information for your data at acceptance. Should your manuscript be accepted for publication, we will hold it until you provide the relevant accession numbers or DOIs necessary to access your data. If you wish to make changes to your Data Availability statement, please describe these changes in your cover letter and we will update your Data Availability statement to reflect the information you provide.

Answer: the GEO Series accession number GSE220161 (https://www.ncbi.nlm.nih.gov/geo/query/acc.cgi?acc=GSE220161) was added in the “Availability of data and materials”.

4. PLOS requires an ORCID iD for the corresponding author in Editorial Manager on papers submitted after December 6th, 2016. Please ensure that you have an ORCID iD and that it is validated in Editorial Manager. To do this, go to ‘Update my Information’ (in the upper left-hand corner of the main menu), and click on the Fetch/Validate link next to the ORCID field. This will take you to the ORCID site and allow you to create a new iD or authenticate a pre-existing iD in Editorial Manager. Please see the following video for instructions on linking an ORCID iD to your Editorial Manager account: https://www.youtube.com/watch?v=_xcclfuvtxQ

Answer: the corresponding author have an ORCID iD and that it is validated in Editorial Manager.

5. Please include captions for your Supporting Information files at the end of your manuscript, and update any in-text citations to match accordingly. Please see our Supporting Information guidelines for more information: http://journals.plos.org/plosone/s/supporting-information.

Answer: the Supporting Information files have been listed at the end of your manuscript.

**Reviewer #1**

Comments to the Author

The paper provides good insights into transcriptional changes occurring in susceptible and resistant sun flower following Sclerotinia sclerotiorum infection. There is a good attempt to measure physiological and correlate it with infection. The authors also provide a good model and graphical abstract as figure 8. For this, I commend the authors. Having said that, my biggest sticking point with the paper is the poor writing. It was difficult to understand some parts of the paper because of the way poor English and grammar. I pointed out a few and suggested revisions, but the whole paper will need to go through a thorough editing process. I was also less impressed by the quality of figures 4 and 5.

Answer: Accepted the suggestion, we have improved the language in this paper. In addition, the figures 4 and 5 were deleted, and these data was listed in S7-8 Tables and Tables 2-3.

Below are my specific comments:

Line 3

S. sclerotiorum

Page 4 line 2

Delete “thus far”

Page 4 line 18-19

Rephrase as follows:

“Both DR genotype C6 and DS genotype B728 are oilseed sunflower inbred lines which were bred by Liaoning Academy of Agricultural Sciences”

Line 23

Italicize “S. Sclerotiorum”

Line 5 Page 5

Replaced “were washing” with “washed”

Page 7 line 2 and throughout the paper,

Italicize “S. sclerotiorum”

Page 8 lines 3-12

Consider rephrasing as follows:

“The physiological response of leaves to S. sclerotiorum inoculation was further evaluated based on the activity of antioxidant enzymes, such as superoxide dismutase activity (SOD), peroxidase (POD), catalase (CAT), glutathione-S-transferase (GST), ascorbate peroxidase (APX) and monodehydroascorbate reductase (MDAR). These data are shown in Table 1. Following infection, the activity of POD, CAT and GST increased significantly in both genotypes, while the SOD activity increased significantly in DR genotype rather than DS genotype. APX and MDAR activity in DS genotype were decreased significantly by S. sclerotiorum infection, while DR genotype showed no significant change. S. sclerotiorum infection, caused higher activity of the enzymes relative to the DS genotype.”

Page 12 line 7

Replace “validate” with “validating”

Page 12 lines 18-24

The sentence in the beginning of the discussion section is poorly written and confusing. Please revise the sentence or consider removing it.

Answer: Accepted the suggestion, we have modified in revised manuscript.

**Reviewer #2:**

The presented study performed pairwise DEG analyses within susceptible and resistant genotypes, resulting in lists of genes and general pathways that may be involved in the sunflower defense response against the pathogen Sclerotinia sclerotiorum. Some main concerns are also listed below:

(1) There are new RNA-seq data generated, but the raw reads are unavailable.

Answer: The raw reads were upload in NCBI, and the GEO Series accession number was GSE220161 (https://www.ncbi.nlm.nih.gov/geo/query/acc.cgi?acc=GSE220161)

(2) The findings are too general, with little information to conduct further studies or cross validations.

Answer: Accepted the suggestion. By RNA-seq analysis, we identified some genes involved in expansins, pectate lyase activities, ethylene biosynthesis and signaling and antioxidant activity, which showed differential expression between DS and DR genotypes. These could potentially be responsible for the differential resistance among two genotypes. However, it was difficulty to identify the candidate genes only via the RNA-seq analysis.

(3) There are expression profiles measured for susceptible and resistant genotypes, but functional/enrichment analyses are mainly focused on DEGs within genotypes. This will mainly lead to functions or pathways that are common between the two different genotypes. Related analyses for uniquely up/down-regulated genes in the resistant genotypes is recommended to identify candidate resistance genes that contribute to the resistance in the DR genotype compared with DS genotype. The analysis described in line 9-16 of page 10 is not comprehensive.

Answer: Accepted the suggestion. The GO analysis for the uniquely up- and down- regulated genes in DR genotype was conducted, and the results were listed in S7 and S8 tables. Also, the top twenty uniquely up- and down- regulated genes in DR genotype was listed in Table 2 and 3, and these functions were annotated. These results were analysized in the revised manuscript.

(4) The data processing parts in the method section needs to be revised to add detail information, including software and parameters used. For example, using "BMKCloud platform" does not provide sufficient information, and MapMan is a software instead of an analysis. A lot of information is missing.

Answer: Accepted the suggestion. We added the detail information in the method section for data processing parts.

(5) The genome assembly information is missing, which is critical for further use and validation of this study. Besides, the gene ID does not sound correct or published ones. For example, "Helianthus_annuus_newGene_2831" in table S2 and "NewGene_11665" in Figure 5B.

Answer: In the transcriptome analysis, it is normal to find new genes, which are not genes in the reference genome, and we will number and annotate the new genes.

Other minor concerns:

(1) There should be a figure for mock-inoculated sample in Figure 1, which only presents inoculated leaves.

Answer: Accepted the suggestion, the figures for mock-inoculated sample were shown in Figure 1.

(2) Nearly a half of expressed genes are differentially expressed, which seems too many. The author may want to double check their data. For example, compare their result to previously published studies.

Answer: We have check our data again. A total of 36969 and 37854 genes were expressed in mock-inoculated samples of DS and DR genotypes, respectively, while 36363 and 39538 in the S. sclerotiorum-inoculated samples.

(3) The reads mapping ratio ranges from 74.8% to 91.8%. This imbalanced mapping ratio may contribute to the super-high number of DEGs.

Answer: In this study, only the mapping ratio of sample of S. sclerotiorum-inoculated B728 was about 70%, which may be caused by the special treatment such as S. sclerotiorum infection.
