# Supplementary material for: Comparative transcriptome analysis in two contrasting genotypes for Sclerotinia sclerotiorum resistance in sunflower
Source: PLoS One. 2024 Dec 19;19(12):e0315458. doi: 10.1371/journal.pone.0315458 (PMC11658501; doi:10.1371/journal.pone.0315458)
Supplement: Attachment — Submitted filename: Response.docx [file pone.0315458.s019.docx]

Reviewer #2:

Please add the NCBI BioProject ID ‘PRJNA908908’ in the manuscript.

Response: All the raw data of the transcriptome sequence have been deposited in the NCBI BioProject database (https://www.ncbi.nlm.nih.gov/bioproject/PRJNA908908) under the BioProject PRJNA908908.

According to figure 4 B-E (figure 6 in previous version), nearly most genes are differentially expressed in both DS and DR genotypes (|log2(FC)| > 1), although the fold change varied. The expression of these genes was not statistically compared between DS and DR genotypes to narrow down the candidates, which could have been inferred from this data.

Response: The expression of these genes was compared between DS and DR genotypes, which were shown in Fig. 4(A-D), and the results section.

The read cleaning method is unclear and irreproducible. For example, a read should not be ‘annotated based on the reference genome’. This revised method section still contains many errors, making the results unreliable.

Response: Raw reads of fastq format were firstly processed through in-house perl scripts. In this step, the clean reads were obtained by removing adapter-containing reads, ploy-N-containing reads, and low quality reads from raw data. The Q20, Q30, GC-content and sequence duplication level of the clean data were calculated. All the downstream analyses were based on clean data with high quality. The adaptor sequences and low-quality sequence reads were removed from the data sets. Raw sequences were transformed into clean reads after data processing. These clean reads were then mapped to the reference genome sequence. Only reads with a perfect match or one mismatch were further analyzed and annotated based on the sunflower reference genome. The HanXRQr1.0 genome was used as reference in the present study. Then, Hisat2 tools soft were used to map with reference genome. The StringTie Reference Annotation Based Transcript (RABT) assembly method was used to construct and identify both known and novel transcripts from Hisat2 alignment results.

The reference genome information is still not provided, making it impossible to assess the results. Not only the ‘new genes’, but also the ‘reference genes’ have only an ‘ID’ available, which largely limits the contribution to the community.

Response: The HanXRQr1.0 genome was used as reference in the present study.

The numbers of expressed genes were previously 22,077 and 22,579, but in the present version, they increased a lot. Again, without the information of the reference genome, it is impossible to know what happened.

Response: After careful inspection again，in the present version, the numbers of expressed genes were false, and those in the previous version were correct.

Without sufficient data, I cannot comment on the numbers listed.

Reviewer #3:

Authors have conducted transcriptome analysis of a resistant and a susceptible genotype of sunflower against Sclerotinia sclerotiorum. I have some suggestions and comments, which are given below. Page numbers are based on the revised, track-change version.

\# Major comments:

1) Figure-4, S1, and S2 are not legible. Hence, I couldn’t check the information associated with these figures. These figures need to be updated.

Response: These figures were modified.

2) One major criticism is that all the follow-up analyses were done with the DEGs (Differentially Expressed Genes) upon infection in both the resistant and the susceptible genotypes. This was pointed out by one of the reviewers. However, the author didn’t do a good job of resolving the issue. Ideally, it would be better to focus on DEGs that are unique to either the resistant or the susceptible genotype. Alternatively, performing clustering of DEGs could have been better.

Response: In GO analysis, the unique up- or down-regulated DEGs in either DS genotype B728 or DR genotype C6.

3) English is very poor in some instances. For example, on page 17, lines 2-3, sentence construction is not correct. The same is true for line-8-9. There are multiple examples like this throughout the manuscript. Correcting those will improve the readability of the manuscript.

Response: According the suggestion, these sentences were modified.

4) Did you use any filtering to get rid of the low-expressed genes, otherwise there will be DEGs that were expressed barely.

Response: Genes with low expression (FPKM <1) will be eliminated by default in the analysis process.

\# Minor comments:

1) Usage of abbreviations should be kept at a minimum level. CWDE for ‘cell wall degrading enzyme’ (page 3, line-11) is unacceptable.

Response: Many abbreviations were deleted in the revised manuscript, such as for oxalic acid, cell-wall-degrading enzymes, programmed cell death, systemic acquired resistance, programmed cell death, pathogenesis related proteins, pathogen-associated molecular pattern.

2) Page-5, line-6, why 24 hpi was used for the experiment?

Response: As the visible lesions appeared in DS genotype rather than DR genotype at 24 h post-inoculation, then the 20 leaves samples within the range of 0.5 cm bordering the extending lesion from inoculated and mock-inoculated plants were harvested for each replicate.

3) Page 6, line 20, how the raw reads were processed?

Response: Raw reads of fastq format were firstly processed through in-house perl scripts. In this step, the clean reads were obtained by removing adapter-containing reads, ploy-N-containing reads, and low quality reads from raw data. The Q20, Q30, GC-content and sequence duplication level of the clean data were calculated. All the downstream analyses were based on clean data with high quality.

4) What kind of analysis was performed using MapMan? MapMan is a software, not an analysis.

Response: According the suggestion, the MapMan analysis was modified as MapMan software.

5) Page 8, line 27, what are the criteria for being expressed?

Response: FPKM>1.

6) GO analysis of all DEGs is meaningless, authors should have focused on unique DEGs.

Response: The GO analysis of all DEGs was deleted in the revised manuscript.

7) Page-9, line-23, “In the CC category, “cell”, “cell part”……were most frequent”. Is this kind of information meaningful?

Response: The GO analysis of all DEGs was deleted in the revised manuscript.

8) Common GOs for both genotypes represented in Figure-3, are not very informative.

Response: The Figure-3 in previous version was deleted, and the S7-8 Tables were added in the present version. The S7-8 Tables were the GO analysis to identify enriched gene ontologies among the common DEGs in DS and DR genotypes.

9) Page 10, lines 11-18, describe GO terms associated with the unique DEGs for each genotype. This section is useful. Authors should expand this section.

Response: According the suggestion, the unique up- and down-regulated DEGs in DS genotype B728 were used for GO analysis, which were also shown in S9-10 Tables.

10) The result section for the analysis using MapMan is very rudimentary.

Response: According another reviewer’s suggestion, in the pathway analysis of DEGs using MapMan, the expressions of these genes involved in plant hormone signaling pathways, redox homeostasis and cell wall organisation were compared between DS and DR genotypes, which were shown in Fig. 4(A-D), and the results section.
