# Supplementary material for: Comparative transcriptome analysis in two contrasting genotypes for Sclerotinia sclerotiorum resistance in sunflower
Source: PLoS One. 2024 Dec 19;19(12):e0315458. doi: 10.1371/journal.pone.0315458 (PMC11658501; doi:10.1371/journal.pone.0315458)
Supplement: Attachment — Submitted filename: Response-R3.docx [file pone.0315458.s021.docx]

Reviewer #1: The ms has improved but still require editorial attention. Please see attached document with comments.

Figures are also low resolution - especially the supplementary figures that show enrichment. Labels on the X-axis have small fonts that are pixelated.

Response: According another reviewer’s suggestion, the manuscript was improved.
